# A stage-specific cancer chemotherapy strategy through flexible combination of reduction-activated charge-conversional core-shell nanoparticles

**DOI:** 10.7150/thno.35057

**Published:** 2019-08-21

**Authors:** Lingfei Han, Yingming Wang, Xiaoxian Huang, Bowen Liu, Lejian Hu, Congyu Ma, Jun Liu, Jingwei Xue, Wei Qu, Fulei Liu, Feng Feng, Wenyuan Liu

**Affiliations:** 1 Department of Pharmaceutical Analysis, China Pharmaceutical University, Nanjing 210009, China; 2 Department of Natural Medicinal Chemistry, China Pharmaceutical University, Nanjing 210009, China; 3 The Joint Laboratory of Chinese Pharmaceutical University and Taian City Central Hospital, Taian City Central Hospital, Taian, 271000, China; 4 Taian City institute of Digestive Disease, Taian City Central Hospital, Taian, 271000, China; 5 Pharmaceutical Department, Taian City Central Hospital, Taian, 271000, China; 6 Jiangsu Food and Pharmaceutical Science College, Huaian 223003, China; 7 Hangzhou Institute of Pharmaceutical Innovation, China Pharmaceutical University, Hangzhou 310018, China

**Keywords:** charge conversion, reduction-activated, tumor penetration, stage-specific chemotherapy, hepatocellular carcinoma

## Abstract

Precision medicine has increased the demand for stage-specific cancer chemotherapy. Drugs with different properties are needed for different stages of tumor development, which is, inducing rapid destruction in the early stage and facilitating deep penetration in the advanced stage. Herein, we report a novel reduction-activated charge-conversional core-shell nanoparticle (CS NP) formula based on ring-closing metathesis of the thiamine disulfide system (TDS) to deliver the chemotherapeutic agent-gambogic acid (GA).

**Methods:** The shell consisted of hyaluronic acid-all-trans retinoid acid with a disulfide bond as the linker (HA-SS-ATRA). The core was selected from poly (γ-glutamic acid) with different grafting rates of the functional group (Fx%) of TDS. GA/C_F100%_S NPs, with the strongest reduction-responsive drug release, and GA/C_F60%_S NPs with the strongest penetration have been finally screened. On this basis, a stage-specific administration strategy against a two-stage hepatocellular carcinoma was proposed.

**Results:** The developed CS NPs have been confirmed as inducing reduction-activated charge conversion from about -25 to +30 mV with up to 95% drug release within 48 h. The administration strategy, GA/C_F100%_S NPs for the early-stage tumor, and sequential administration of GA/C_F60%_S NPs followed by GA/C_F100%_S NPs for the advanced-stage tumor, achieved excellent tumor inhibition rates of 93.86±2.94% and 90.76±6.43%, respectively.

**Conclusions:** Our CS NPs provide a novel platform for charge conversion activated by reduction. The stage-specific administration strategy showed great promise for cancer therapy.

## Introduction

Chemotherapy still plays an important role in cancer treatment, despite the emergence of various promising alternatives, such as photodynamic and photothermal therapy 1,2. In the context of precision medicine 3, achieving optimal efficacy at each stage of a cancer is of prime concern, and this gives rise to the concept of stage-specific cancer therapy 4. Generally, an early-stage tumor develops quickly due to sufficient nutrition supplies from adjacent blood vessels 5. Hence, efficient tumor targeting and rapid release of high doses of drugs are required to swiftly destroy the fast-growing tumor 6, which has been widely realized through the use of various stimuli-responsive drug carriers 7. For an advanced-stage tumor, however, tumor penetration becomes another issue of concern 8,9. This is because internal hypoxia becomes more prevalent with increasing tumor volume, which could promote the proliferation of cancer stem cells. Such cells are particularly aggressive and can lead to metastasis and relapses 10-12. In addition, it is difficult for drugs to access deep sites of an advanced-stage tumor of large size with dense stroma due to several biological barriers 13. Worse still, the deep tumor cells could gradually acquire resistance due to long-term exposure to a sublethal concentration of the therapeutic agent and ultimately become refractory 14,15. Thus, developing a drug carrier with improved penetration ability would be significant for enhanced cancer treatment efficacy.

Many researchers have addressed this issue through different strategies, including size switching 15,16, charge conversion 17,18, modifications with penetration-promoting ligands 19,20 and modulations of the tumor microenvironment 21,22. Among these approaches, charge conversion, usually triggered by various stimuli, such as pH, reactive oxygen species, protease, heat or light 17, provides an effective means of penetrating deep into tumor cells. In general, nanoparticles with positively charged surfaces can be effectively internalized by cells through electrostatic interactions with the negatively charged cell plasma membrane 23, whereas cationic carriers in the bloodstream show strong non-specific cellular uptake and can cause severe serum inhibition 24,25. Given that, it is necessary to design charge-conversional drug carriers to address issues of both safety and penetration. Unfortunately, however, reduction-activated charge conversion has rarely been reported. Glutathione (GSH), as the most common biological reducing substance, is present at a high concentration in tumor cells (2-10 mM) 26 and has been widely exploited in many rapid drug release carriers through cleavage of a disulfide bond. Therefore, we sought to construct a charge-conversional carrier induced by the cleavage of a disulfide bond.

Fortunately, a brain-targeting strategy using TDS termed “lock-in”, aimed at blood-brain barrier (BBB), gave us inspiration 27. TDS is a *cis*-2-formylamino-ethenyl-thio derivative and has been clinically used as a fat-soluble prodrug of thiamine (Vitamin B1) 28,29. On entering the BBB, TDS is reduced by GSH and undergoes ring-closing to form a thiazolium salt, which is lipophobic and cannot cross the BBB *via* diffusion, thus becoming “locked” in the brain and providing sustained release of the drug through hydrolysis 30,31. However, TDS also readily enters red blood cells rich in GSH and hemoglobin 32,33, leading to its undesirable distribution and stability in the bloodstream. Further research indicated that utilization of only the 4-methyl-5-hydroxyethyl thiazole derived from TDS could overcome these drawbacks 27,34.

**Scheme 1:**
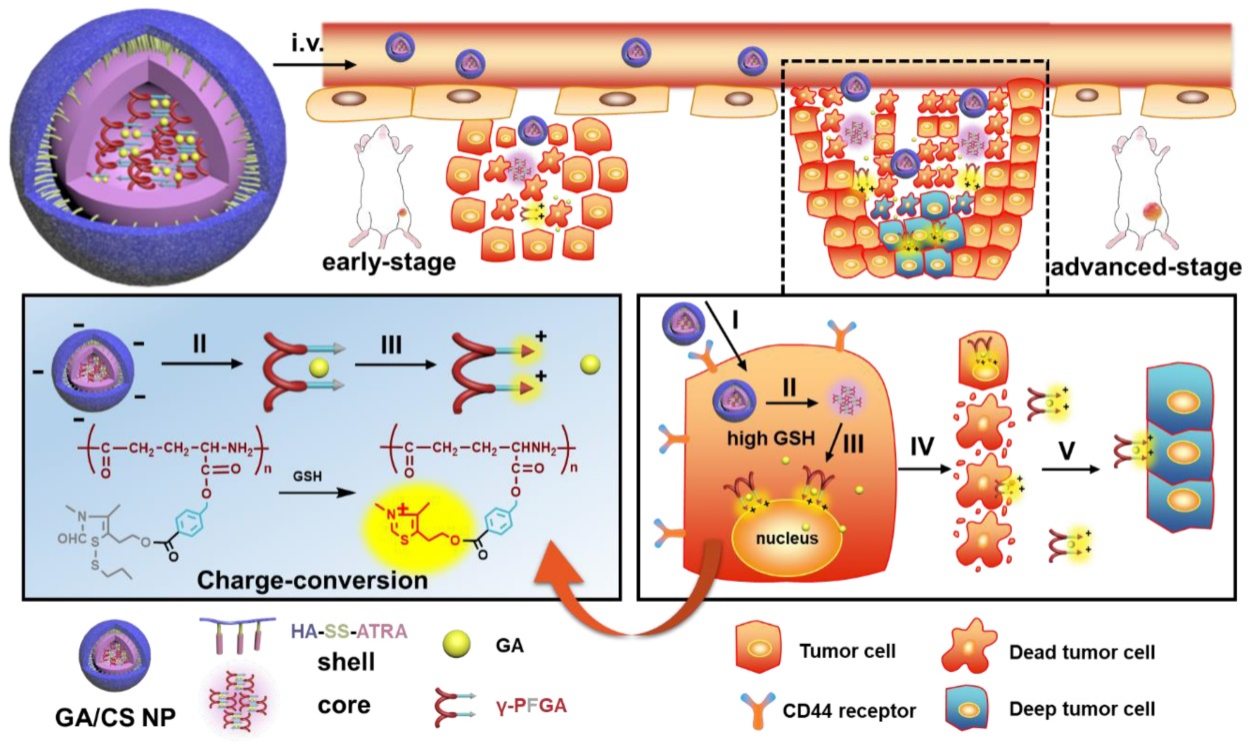
The design and drug delivery of the functional GA/CS NPs for a two-stage cancer therapy. The reduction-activated charge-conversional CS NPs were composed of HA-SS-ATRA as shell and GA-loaded γ-PFGA as core. After leakage from the tumor blood vessels, it first achieved the superficial zone. Take the advanced-stage tumor as an example, I) HA-CD44 receptor mediated endocytosis, II) reduction-activated degradation of the shell material, III) reduction-activated degradation of the core material and consequent charge conversion and nuclei targeting due to the ring-closing metathesis of the functional group, IV) escape of the positively charged core material with remaining drugs from the dead tumor cells, V) electrostatic-attraction-mediated deep tumor penetration.

In view of the above findings, in this study, a novel reduction-activated charge-conversional core-shell nanoparticle (CS NP) formula based on TDS as the functional group (FG) has been designed for rapid drug release and deep penetration in cancer treatment (Scheme 1). It comprised HA-SS-ATRA as the shell (constructed as described in our previous study 35), poly (γ-glutamic acid) with different grafting rates of the FG (γ-PFGA, using the grafting-to method) as the core and GA as the chemotherapeutic agent. The HA-coated GA/CS NPs were negatively charged in blood circulation and accumulated in tumor sites through an enhanced permeability and retention (EPR) effect 36. They were then endocytosed through HA-CD44 receptor interaction 37 and achieved the degradation of the shell and core by intracellular GSH. Finally, GA-loaded γ-PFGA acquired electrostatic-attraction-mediated nuclei-targeting and deep tumor penetration abilities owing to the positive charge generated from ring-closing metathesis of the FG 38. GA is a multi-target drug, and is involved in many mechanisms related to nuclei 39-41. Notably, although fast degradation of the materials would lead to rapid drug release, less of the drugs would be left for penetration into deep tumor cells. Therefore, four types of GA/CS NPs (grafting rate of FG: 0%, 30%, 60% and 100%), exhibiting different properties, were screened for the most powerful antitumor efficacy (the rapidest drug release, GA/C_F100%_S NP) and the strongest penetration ability (GA/C_F60%_S NP) respectively.

**Scheme 2:**
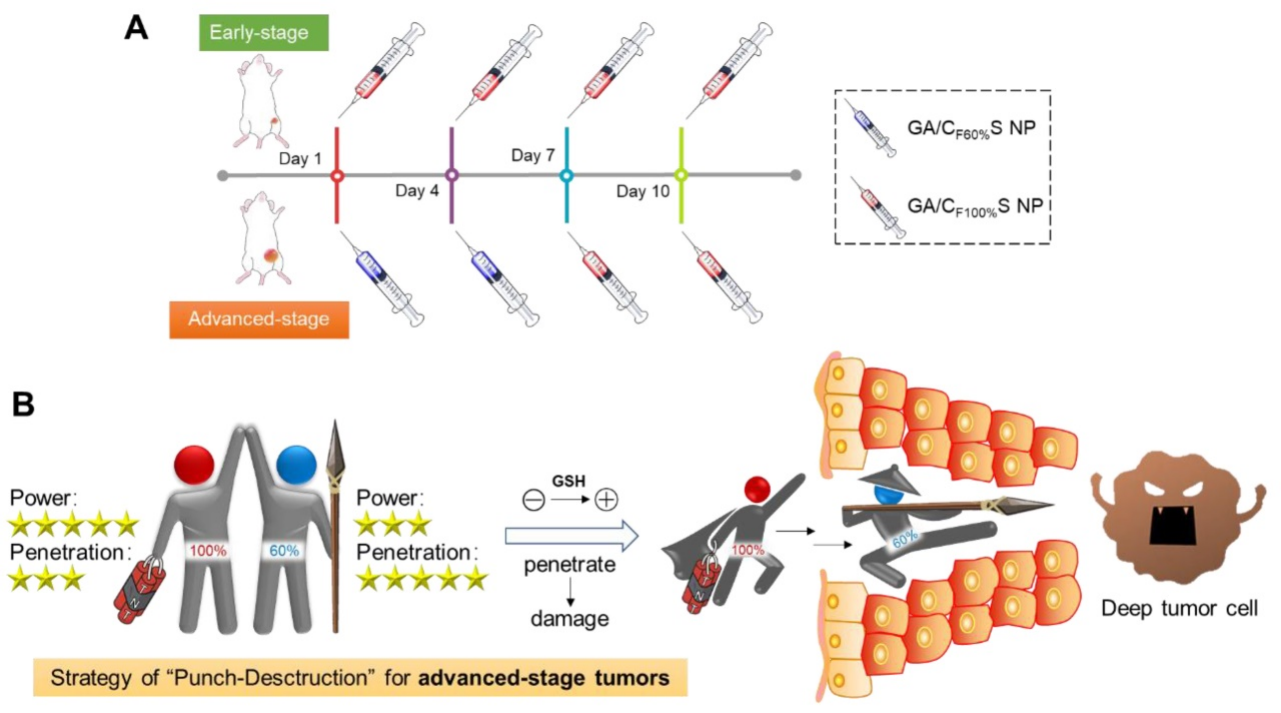
** (A)** Stage-specific administration strategy for a two-stage hepatocellular carcinoma: GA/C_F100%_S NPs for the early-stage tumor and sequential administration of GA/C_F60%_S NPs followed by GA/C_F100%_S NPs for the advanced-stage tumor. **(B)** Schematic of “Punch-Destruction” strategy for the advanced-stage tumor treatment. First, the GA/C_F60%_S NPs with the strongest drug-loaded penetration ability make a hole to reach deep tumor cell; then the GA/C_F100%_S NPs, with the strongest destruction power due to the rapidest reduction-responsive drug release, kill the remaining tumor cells along the carved-out route.

To further confirm the functionality of our GA/CS NPs and embody our understanding of precision medicine, a two-stage hepatocellular carcinoma model (Heps) was established according to the Tumor-Node-Metastasis (TNM) staging criteria 42, including early and advanced-stages. Meanwhile, tumor biomarkers, as one of the cornerstones for precision medicine 43, were also utilized to assist establishing the model. On this basis, we proposed a stage-specific administration strategy. In brief, for early-stage tumors, GA/C_F100%_S NPs were the best choice to exert a direct and strong damaging effect due to rapid reduction-responsive drug release. For advanced-stage tumors, sequential administration of GA/C_F60%_S NPs followed by GA/C_F100%_S NPs, termed the “Punch-Destruction” strategy, has been conducted for the first time. First, GA/C_F60%_S NPs, with the strongest drug-loaded penetration and medium reduction-responsive drug release ability, opened fissures to reach deep tumor cells, and then GA/C_F100%_S NPs, with the strongest reduction-responsive drug release ability, killed the remaining tumor cells along the carved-out route (Scheme 2). All the results indicated that proper utilization and flexible combination of these novel reduction-activated charge-conversional CS NPs could serve as an effective strategy for stage-specific treatment for enhanced antitumor efficacy.

## Methods

### Materials

Poly (L-glutamic acid) (γ-PGA-Na) (10 kDa) was purchased from Nanjing Shineking Biotech Co., Ltd. (Jiangsu, China). Cetyltrimethylammonium bromide (CTA-Br) was purchased from Sinopharm Chemical Reagent Co., Ltd. (Shanghai, China). Iodomethane, 4-methyl-5-hydroxyethyl thiazole, 1-bromopropane, benzyl bromide, bromomethyl benzoic acid, and fluorescein isothiocyanate (FITC) were purchased from Sigma-Aldrich (St. Louis, MO, USA). All of the organic solvents used, such as dimethyl sulfoxide (DMSO), ethanol, ethyl acetate, and *N*,*N*-dimethylformamide (DMF), were purchased from Nanjing Chemical Reagent Co., Ltd. (Nanjing, China). Distilled water was purified by a Millipore Milli Q-Plus system (Millipore, Bedford, MA, USA).

### Synthesis of the shell material

The HA-SS-ATRA conjugate was synthesized following the methods devised in our previous research 35. In brief, HA-Na (14.6 kDa, 24.4 mmol) and tetra-*n*-butylammonium hydroxide (TBA, 19.4 mmol) were combined in distilled water (60 mL), and the solution was stirred for 1 h to generate HA-TBA. ATRA (17.18 mmol), triethylamine (18.90 mmol), and bis(2-bromoethyl) disulfide (17.18 mmol) were mixed in THF (70 mL) by stirring at room temperature for 3 h to afford ATRA-bromoethyl disulfide. HA-TBA and ATRA-bromoethyl disulfide (molar ratio 5:2) were then stirred in DMSO for 48 h at 50ºC. Subsequent dialysis with NaCl solution (5%, *w/v*) and distilled water afforded HA-SS-ATRA conjugate as the shell material. Furthermore, FITC-labeled HA-SS-ATRA was synthesized following the reported method 44. In brief, HA-SS-ATRA (100 mg) was dissolved in distilled water (50 mL), and then a solution of FITC (20 mg) in ethanol (2 mL) was added dropwise. The mixture was agitated in the dark for 3 days. The FITC-labeled HA-SS-ATRA was recovered after dialysis with water for 2 days and then freeze-dried.

### Synthesis of the core material (Figure 1A)

#### 1 Synthesis of γ-PGA-CTA

The γ-PGA-Na (10 kDa, 10 g) was dissolved in distilled water (200 mL) under vigorous stirring at 40°C. Aqueous CTA-Br solution (3%, *w/v*) was then added dropwise. The white precipitate of γ-PGA-CTA formed was collected by filtration.

#### 2 Synthesis of 3,4-dimethyl-5-hydroxyethyl thiazolium iodide (compound **1**)

CH_3_I (106.02 mmol) was added dropwise to 4-methyl-5-hydroxyethyl thiazole (35.19 mmol) in an autoclave at 60ºC over a period of 3 h. After distillation under reduced pressure, the brown solid product was washed with diethyl ether (yield 100%). ^1^H NMR (300 MHz, CD_3_OD, *δ*): 2.54 (s, 3H, 4-C**H**_3_), 3.13 (t, 2H, *J*=6 Hz, -C**H**_2_CH_2_OH), 3.83 (t, 2H, *J*=6 Hz, -CH_2_C**H**_2_OH), 4.16 (s, 3H, 2-C**H**_3_), 9.89 ppm (s, 1H, 2-**H**); MS (*m*/*z*): 158 [M+H]^+^ (Figure S1).

#### 3 Synthesis of propyl Bunte salt (compound **2**)

The 1-bromopropane (49.83 mmol) was dissolved in ethanol (30 mL) containing PEG400 (0.2 mL). A solution of Na_2_S_2_O_3_**·**5H_2_O (45.21 mmol) in water (10 mL) containing PEG400 (0.1 mL) was then added dropwise and the mixture was heated under reflux for 7 h at 69ºC. The product was obtained as a white microcrystalline powder after distillation under reduced pressure (yield 100%). ^1^H NMR (300 MHz, D_2_O, *δ*): 0.86 (t, 3H, *J*=6 Hz, -SCH_2_CH_2_C**H**_3_), 1.64 (m, 2H, -SCH_2_C**H**_2_CH_3_), 2.95 ppm (t, 2H, *J*=6 Hz, -SC**H**_2_CH_2_CH_3_); MS (*m*/*z*): 155 [M-H]^-^ (Figure S2).

#### 4 Synthesis of 3,4-dimethyl-5-hydroxyethyl thiazolium iodide (ring-opening) with propyl (compound **3**)

Aqueous NaOH (1 g/mL, 127.5 mmol) was added to a stirred solution of compound **2** (21.1 mmol) in water (16 mL) under argon over a period of 30 min with cooling in ice, and then ethyl acetate (60 mL) was added over 10 min. A solution of compound **3** (52.5 mmol) in water was added to the above system over a period of 2 h. After separating the layers, the ethyl acetate phase was concentrated under reduced pressure. The concentrate was purified by column chromatography on silica, eluting with CH_2_Cl_2_/MeOH (50:1, v/v). Yield: 79.85%; ^1^H NMR (300 MHz, CDCl_3_, *δ*): 0.95 (t, 3H, *J*=7.5 Hz, SCH_2_CH_2_C**H**_3_), 1.65 (m, 2H, SCH_2_C**H**_2_CH_3_), 1.95/1.99 (s, 3H, 4-C**H**_3_), 2.57 (t, 2H, *J*=6 Hz, -C**H**_2_CH_2_OH), 2.85 (t, 2H, *J*=6 Hz, SC**H**_2_CH_2_CH_3_), 2.93/3.04 (s, 3H, 3-C**H**_3_), 3.77 (t, 2H, *J*=6 Hz, -CH_2_C**H**_2_OH), 5.29 (s, 1H, -O**H**), 7.90/7.94 ppm (s, 1H, -C**H**O); MS (*m*/*z*): 272 [M+Na]^+^ (Figure S3).

#### 5 Synthesis of 3,4-dimethyl-5-hydroxyethyl thiazolium iodide (ring-opening) with propyl* p*-bromomethyl benzoate (compound **4**, functional group)

The 4-bromomethyl benzoic acid (32.26 mmol), EDCI (35.75 mmol), and DMAP (16.92 mmol) were mixed in CH_2_Cl_2_ (120 mL) and DMF (50 mL). The mixture was stirred for 30 min with cooling in ice. A solution of compound 4 (20.05 mmol) in CH_2_Cl_2_ (80 mL) was added over a period of 48 h at room temperature. After completion of the reaction, the CH_2_Cl_2_ was evaporated under reduced pressure, the remaining solution was poured into water (150 mL), and the mixture was extracted with ethyl acetate (3×200 mL). The ethyl acetate extracts were combined, washed with water (3×600 mL), dried over anhydrous sodium sulfate, and concentrated. The obtained pale-yellow solid was purified on a silica gel column, eluting with petroleum ether/ethyl acetate (3:1, *v/v*) to afford the product as a yellow oil (compound 4, functional group). Yield: 22.05%; ^1^H NMR (300 MHz, CDCl_3_, *δ*): 0.97 (t, 3H, *J*=7.5 Hz, SCH_2_CH_2_C**H**_3_), 1.68 (m, 2H, SCH_2_C**H**_2_CH_3_), 1.96/1.98 (s, 3H, 4-C**H**_3_), 2.61 (t, 2H, *J*=6 Hz, -C**H**_2_CH_2_OH), 2.93/3.02 (s, 3H, 3-C**H**_3_), 3.09 (t, 2H, *J*=6 Hz, SC**H**_2_CH_2_CH_3_), 4.52 (t, 2H, *J*=6 Hz, -CH_2_C**H**_2_OH), 4.62 (s, 2H, Ph-C**H**_2_Br), 7.48 (d, 2H, *J*=9 Hz, Ph-3,5-**H**), 7.88 (s, 1H, -C**H**O), 8.01 ppm (d, 2H, *J*=9 Hz, Ph-2,6-**H**); MS (*m*/*z*): 446 [M+H]^+^ (Figure S4).

#### 6 Synthesis of γ-PFGA with different grafting rates of the functional group

The γ-PGA-CTA (0.962 mmol) was first dissolved in NMP under stirring. NaHCO_3_ (0.39 g), benzyl bromide, and compound **4** were then added to the γ-PGA-CTA solution at four different molar ratios, specifically 0%, 30%, 60%, and 100% (total 1 mmol). The reaction system was stirred for 8 h at 50°C. The mixture was then cooled to room temperature and filtered to remove the insoluble precipitate. The supernatant was poured into pre-cooled methanol/water (1:1, *v/v*; 60 mL), containing 0.5% HCl, and stirring was continued for 0.5 h. The precipitate was collected by filtration and dried under vacuum to obtain four types of core materials, namely 0%, 30%, 60%, and 100% γ-PFGA. The degree of grafting rates was determined by ^1^H NMR spectrometry.

### Preparation of CS NPs and GA/CS NPs with different grafting rates of the functional group

The γ-PFGA-HA-SS-ATRA core-shell nanoparticles (CS NPs) were prepared by a one-step method. Briefly, γ-PFGA (0%, 30%, 60%, 100%) was dissolved in DMSO at 1.5 mg/mL, with or without GA at 0.15 mg/mL, as the organic phase. HA-SS-ATRA was dispersed in water at 0.15 mg/mL as the aqueous phase and heated to 65°C prior to preparation of the CS NPs. The organic phase was then added to the preheated aqueous phase (0.5 mL/min) under vigorous stirring. The temperature of the mixture was kept at 65°C during nanoprecipitation, and the nanoparticles were allowed to self-assemble for 2 h at room temperature. The remaining organic solvent and free molecules were removed by threefold passage of the solution of CS NPs through an Amicon Ultra-15 centrifugal filter (Millipore, Billerica, MA, USA) with a molecular weight cut-off of 100 kDa. The CS NPs were then resuspended in water to obtain the desired final concentration, and the suspension was stored at 4°C prior to use. The size, polydispersity index (PDI), and zeta potential were measured by means of a Malvern Zetasizer Nano-ZS90 (Malvern Instruments, UK) and a transmission electron microscope (TEM, Hitachi HT7700). After extraction with a five-fold volume of methanol for 5 min and filtration through a 0.22 μm membrane, the drug encapsulation efficiency (EE%=drug encapsulated in nanoparticles/initial drug input × 100%), drug-loading capacity (DL%=drug encapsulated in nanoparticles/(drug + initial material input) × 100%), drug release profile, and * in vitro* stability were determined by HPLC (Shimadzu 20AT).

### * In vitro* drug release in PBS and GSH

Aliquots (2 mL) of suspensions of different types of GA/CS NPs (1 mg/mL) containing 0.1% (*w/v*) Tween 80 were placed in dialysis bags (molecular weight cut-off 3500 Da). They were then dialyzed with PBS (100 mL) containing 0.1% (*w/v*) Tween 80 (with or without 10 mM GSH), and the medium was replaced every 12 h. At predetermined time points, aliquots (50 μL) of the samples in the dialysis bags were removed for HPLC analysis. Drug release curves of the GA/CS NPs in PBS and GSH were plotted to assess the release efficacy in reducing environments.

### Reduction-responsive degradation

Aliquots (900 µL) of suspensions of different types of CS NPs (100 μg/mL) were mixed with aliquots (100 µL) of GSH solution (100 mM) and incubated at 37°C. At predetermined time points, the size distribution and morphology were measured by dynamic light-scattering (DLS) and TEM.

### Reduction-triggered charge conversion

Aliquots (900 µL) of suspensions of NPs prepared with different core materials (100 μg/mL) were mixed with aliquots (100 µL) of DTT solution (100 mM) and incubated at 37°C. At predetermined time points, the zeta potential was measured by DLS. Further, adequate amount of different types of core materials was treated in the same way. The reaction was terminated by adding an equivalent of H_2_O_2_ at a predetermined time point, and the mixture was dialyzed. After work-up, the product was freeze-dried for structure confirmation of its charge conversion by ^1^H NMR spectrometry.

### Cell culture

Human hepatocellular carcinoma cell line HepG2, murine hepatocellular carcinoma cell line Heps, and human hepatocyte cells (L02) were obtained from the cell bank of the Chinese Academy of Sciences (Shanghai, China). Cells were cultured in 1640 medium containing 10% FBS, 100 U/mL penicillin, and 100 μg/mL streptomycin and maintained at 37°C in a humidified atmosphere containing 5% CO_2_ in an incubator.

### Cytotoxicity

L02 and HepG2 cells were seeded in 96-well plates at a density of 5×10^3^ cells/well. After incubation for 24 h, the cells were separately treated with various concentrations of different types of CS/NPs, GA/CS NPs, and GA for 24 h. Each type of GA/CS NPs was pretreated with 50 μM BSO, 10 mM GSH, and 2 mg/mL HA for 12 h, 2 h, and 2 h, respectively, to investigate the enhanced cytotoxicity due to reduction-sensitivity and HA-CD44-mediated endocytosis. After incubation, 5 mg/mL MTT solution (20 μL/well) was added and the mixtures were cultured for a further 4 h. The supernatant was discarded and DMSO was added (150 μL/well). The absorbance at 492 nm was then measured by means of a microplate reader (Thermo Electron Corporation). Untreated cells were used as a 100% viability control and triplicate determinations were performed for each treatment.

### Cell apoptosis assay

Apoptosis of HepG2 cells was detected using an Annexin V-FITC apoptosis detection kit (BD Biosciences). The cells (5 × 10^4^ per well) were seeded in 12-well plates and cultured for 24 h. For Annexin V-FITC assay, the cells were incubated with different types of GA/CS NPs and GA (1 μg/mL) for 24 h. The subsequent procedures were performed in accordance with the manufacturer's protocols. Finally, the cells were analyzed by flow cytometry (Miltenyi Biotec, Germany) and data were processed using FlowJo software.

### Cellular uptake

The cellular uptake behaviors of the nanoparticles were also studied in HepG2 cells by loading coumarin-6 (C6; Sigma-Aldrich) instead of GA and monitoring by flow cytometry (FCM) and confocal laser scanning microscopy (CLSM). HepG2 cells were seeded in 35 mm confocal microscopy dishes at a density of 1×10^5^ cells/well and incubated for 24 h. The medium in each well was then replaced with the same concentration of free C6 or C6/CS NPs for 0.5, 2, or 4 h at 37°C. For the 4 h group, another group of 2 mg/mL HA was pretreated for 2 h. The cells were then washed twice with PBS and fixed with 4% paraformaldehyde for 15 min. DAPI was then added to stain the cell nuclei. Fluorescence images were acquired by means of an LSM 800 laser confocal scanning microscope (Zeiss, Germany). The images were analyzed using Zeiss CLSM software. For quantitative analysis, the cells were seeded in a 12-well plate (1 × 10^5^ cells/well) and incubated for 24 h. Free C6 and C6/CS NPs were then added to each well. For the 2 h group, another group of 2 mg/mL HA was pretreated for 2 h. After incubation at 37°C for 0.5 h, 2 h, or 4 h, the cells were digested with 0.25% (w/v) trypsin, washed thrice with PBS, and then resuspended in PBS (500 μL). Fluorescence histograms were then recorded by FCM and analyzed using FlowJo software.

### Nuclei targeting

The method was the same as that described for cellular uptake. For each C6/CS NP group incubated for 4 h, samples were pretreated with 50 μM BSO and 10 mM GSH for 12 h and 2 h, respectively.

### Intracellular shell detachment

The method was the same as that described for cellular uptake. 1,1-Dioctadecyl-3,3,3,3-tetramethylindotricarbocyanine iodide (DiR, a near-infrared dye; Thermo Fisher Scientific, USA) was used as a fluorescent probe. HepG2 cells were incubated with (GA+DiR)/C_F60%_S NPs using FITC-labeled HA-SS-ATRA for 1 h or 4 h.

### * In vitro* two-dimensional intercellular delivery

HepG2 cells seeded on coverslips were pretreated with (GA+DiR)/C_F60%_S NPs or free GA+DiR at a GA concentration of 4 μg/mL for 6 h. The pretreated cells (A) were withdrawn and co-incubated with fresh cells on a coverslip (B) for 12 h in fresh culture medium. B was then withdrawn and co-incubated with fresh cells on another new coverslip (C) for 12 h in fresh culture medium. The cells were subsequently washed thrice with ice-cold PBS and stained with propidium iodide (PI) (20 μg/mL) for 15 min. All cells (A, B, and C) were washed thrice with ice-cold PBS and observed by means of CLSM.

### * In vitro* tumor penetration in 3D tumor spheroids

*In vitro* 3D tumor spheroids of HepG2 cells were developed by a liquid overlay method 45. Uniform and compact tumor spheroids were selected for subsequent studies. The tumor spheroids were incubated with different types of (GA+C6)/CS NPs and GA+C6 at a GA concentration of 4 μg/mL, each for 6 h. The tumor spheroids were then washed thrice with ice-cold PBS, fixed with paraformaldehyde in PBS (4%, w/v) for 15 min, and placed in cavity microscope slides. Images of the tumor spheroids were acquired by Tomoscan with Z-stack imaging at 10 μm intervals from the top of the spheroid to the middle by CLSM. Semi-quantitative analysis of the mean fluorescence intensity was conducted with Image Pro Plus.

### Animals and tumor xenograft models

Male ICR mice (18-22 g) were purchased from the Medical Center of Yangzhou University (Yangzhou, China). All of the animals were pathogen-free and were allowed free access to food and water. The experiments were carried out in compliance with the National Institute of Health Guide for the Care and Use of Laboratory Animals of China Pharmaceutical University and approved by the Animal Ethics Committee of China Pharmaceutical University. To set up the tumor xenograft model, 1×10^6^ Heps cells were subcutaneously injected into the left leg of each ICR mouse. Tumor volume (V) was determined by measuring length (L) and width (W), and calculated as V = 0.5×L×W^2^.

### *In vivo* tumor targetability

An IVIS spectrometer (Perkin-Elmer, USA) was used to monitor the biodistribution of CS NPs in the tumor-bearing mice. Six tumor-bearing mice were randomly divided into two groups, the members of which were intravenously injected with the same dose of DiR (0.1 mg/kg) in the form of free DiR solution, DiR-loaded C_F60%_S NPs, and DiR-loaded C_F100%_S NPs, respectively. Imaging was performed at 8 h after injection to estimate the time-correlated excretion profile. Region of interest (ROI) image analysis was performed using Living Image 4.5 software.

### *In vivo* vascular drug distribution of intravenously injected GA/CS NPs in tumor-bearing mice

When the tumor volume reached 300 mm^3^, the tumor-bearing ICR normal mice were intravenously injected with DiR, DiR/C_F60%_S NPs, and DiR/C_F100%_S NPs at the same dose of DiR (0.1 mg/kg). At 24 h post-injection, tumors were collected, washed with PBS, and examined by using cryotome sections. Frozen tumor sections were stained with FITC-CD31 antibodies (Biotech Laboratories, USA) at 37ºC for 60 min, thrice washed with PBS, and observed by means of CLSM.

### *In vivo* tumor penetration of intratumorally injected GA/CS NPs in tumor-bearing mice

Tumor-bearing ICR normal mice were intratumorally injected with GA+DiR, GA+DiR/C_F60%_S NPs, or GA+DiR/C_F100%_S NPs at fixed doses of GA (6 mg/kg) and DiR (0.1 mg/kg) at a consistent depth of needle insertion (2 mm below the tumor surface). At 24 h post-injection, tumors were collected, washed with PBS, and sliced into different layers from the top to the middle by cryotome sectioning. The nuclei of the tumor cells were stained with Hoechst 33258 (1 μg/mL) for 30 min. The tumor sections were observed by CLSM. Semi-quantitative analysis of the mean fluorescence intensity was conducted with Image Pro Plus software.

### *In vivo* anti-tumor activity

1) Early-stage model: The * in vivo* anti-tumor activity study was started when tumor volume in the mice reached around 100 mm^3^. 2) Advanced-stage model: The * in vivo* anti-tumor activity study was started when tumor volume in the mice reached around 1000 mm^3^. Heps xenograft tumor-bearing mice were randomly divided into five groups (*n*=6): saline, GA/IS (Injection solution), GA/C_F60%_S NPs, GA/C_F100%_S NPs, and GA/C_F60%_S NPs + GA/C_F100%_S NPs. Each group was intravenously injected with the different formulations at a dose of 6 mg/kg every 3 days. All animals were dosed four times, and the first dose day was set as 0 d. The tumor volumes and body weights of the mice were monitored every other day. At the prescribed time (14 d), the tumors and other immune organs (liver, spleen, and thymus) were harvested and weighed. The tumor inhibition rate was calculated using the formula: TIR% = (W_c_-W_t_)/W_c_×100% (W_c_: tumor weight of the control group; W_e_: tumor weight of the treatment group). For H&E staining, the tumors were washed and fixed in 4% formaldehyde solution and then embedded in paraffin blocks. For each type of tumor model (early stage, advanced stage, and treatment groups), cell proliferation was determined by EdU assay (BeyoClick), and the biomarker of hepatocellular carcinoma (AFP) was determined by ELISA (EIAab) of the tumor homogenate.

### Statistical analysis

Data are given as mean ± standard deviation. Statistical significance was tested by a two-tailed Student's *t*-test. Statistical significance was set at ^*^p < 0.05, ^**^p < 0.01, and ^***^p < 0.001.

## Results and discussion

### Synthesis and preparation of functional CS NPs

**Figure 1:**
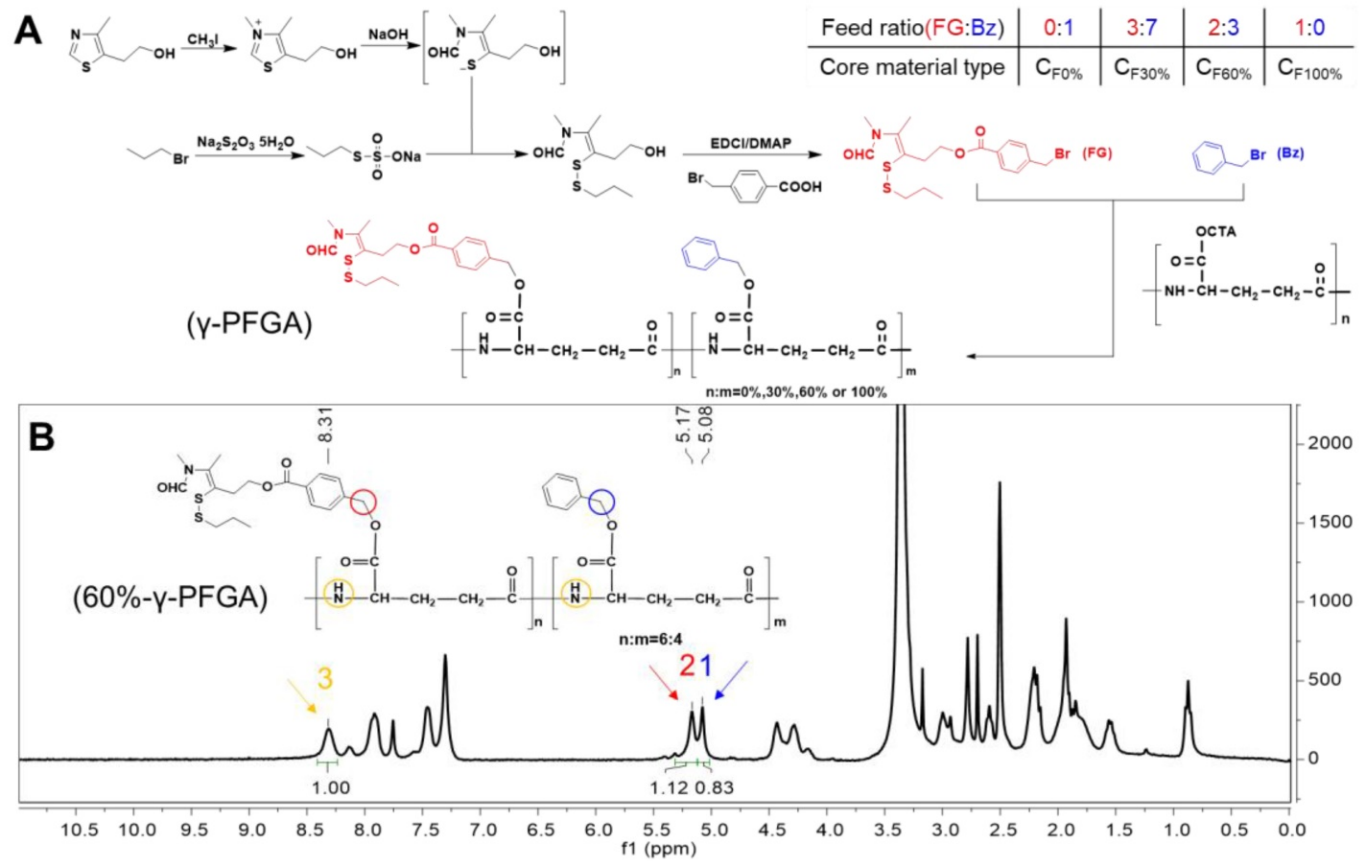
** (A)** Synthesis of different types of γ-PFGA. **(B)** ^1^H-NMR spectrum of 60%-γ-PFGA.

The functional CS NPs were composed of core and shell materials. The core material, with different grafting rates of FG (TDS), was synthesized from 4-methyl-5-hydroxyethyl thiazole, benzyl bromide and γ-PGA (Figure 1A). The γ-PGA is a water-soluble polymer, and so would not react readily with water-insoluble benzyl bromide and FG. To overcome this issue, we synthesized γ-PGA-CTA to facilitate a homogeneous reaction. Through controlling the feed ratio of FG and benzyl (Bz), we obtained different grafting rates of γ-PFGA, including 0%, 30%, 60% and 100% -γ-PFGA, as characterized by ^1^H-NMR (Figure S5). To calculate the actual grafting rate of FG, considering 60%-γ-PFGA as an example (Figure 1B), peak 1(δ=5.08 ppm, C**H**_2_Ar), peak 2 (δ=5.17 ppm, PhC**H**_2_) and peak 3 (δ=8.31 ppm, N**H**) were used as markers for Bz, FG and γ-PGA, respectively. The area ratio of peak 2 / (peak 2 + peak 1) was calculated as the grafting rate. The calculated grafting rate of 60%-γ-PFGA was 57.44%, within the margin of error. The results of similar calculations on other types of γ-PFGA are listed in Table S1. With the grafting rate of FG increasing, the core material will show stronger reduction-sensitivity and charge-conversion ability. Our prime concern was to select the best type of core material for different goals. The shell material, HA-SS-ATRA, was synthesized according to the method described in our previous study 35.

**Figure 2:**
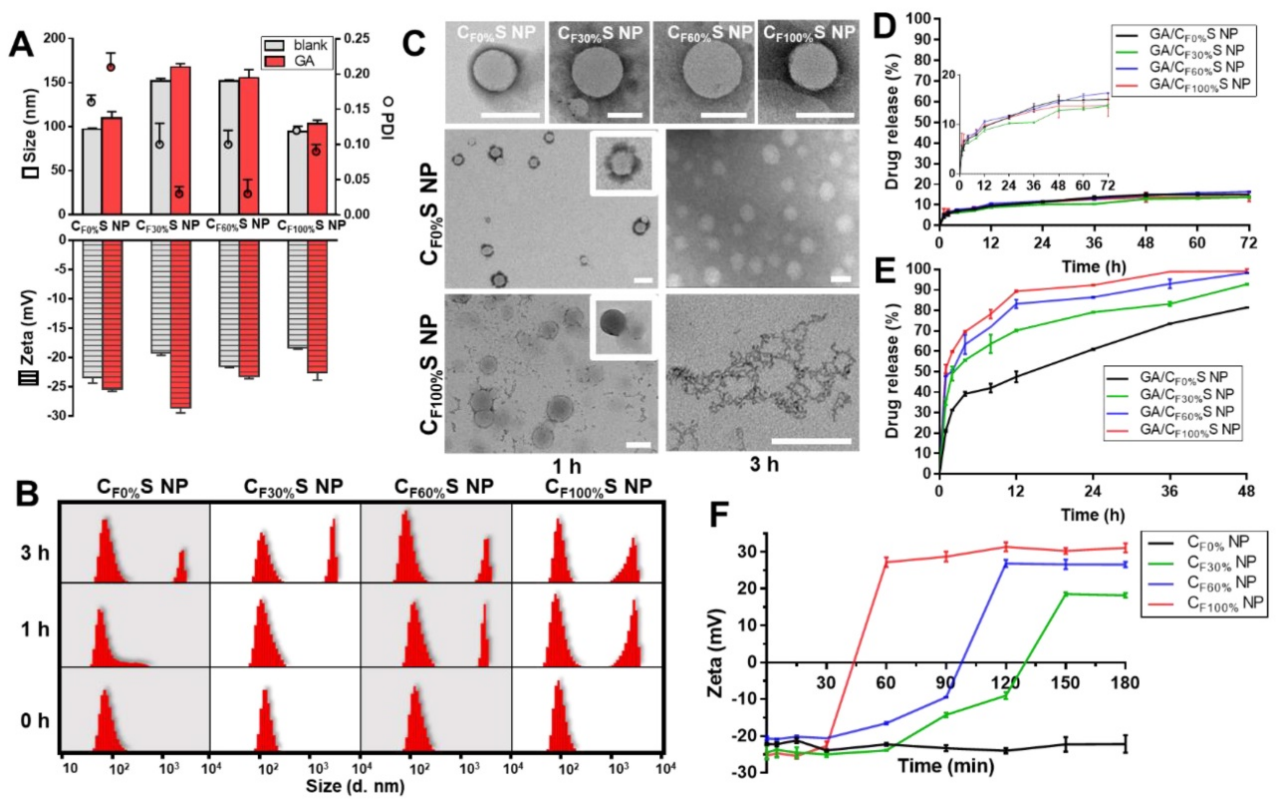
** (A)** Size, PDI and zeta potential of different types of blank and GA-loaded functional CS NPs. **(B)** Size distribution of different types of CS NPs incubated with 10 mM GSH for 0, 1 and 3 h by DLS. **(C)** Morphology changes of different types of CS NPs incubated with 10 mM GSH for 1 and 3 h by TEM. Scale bars indicate 100 nm. **(D)** & **(E)** *in vitro* drug release profiles of different types of GA/CS NPs in PBS **(D)** and 10 mM GSH **(E)**. **(F)** Changes in the zeta potential of different types of core materials NP incubated with 10 mM DTT over time.

Different types of functional CS NPs were prepared by the “one step method” optimized in our recent work 46. Size, polydispersity index (PDI), zeta potential, EE% and DL% were summarized in Figure 2A and Table S2. Blank CS NPs showed a small size (94.30±6.10 nm to 151.90±2.83 nm) and negative zeta potential (-23.50±0.85 mV to -18.30±0.28 mV) due to π-π stacking interactions of the core 47 and the HA-coated shell. Notably, the C_F30%_S and C_F60%_S NPs were about 50 nm larger than the C_F0%_S and C_F100%_S NPs, which may result from the uniformity of the core material. The more uniform the material is, the more readily it could form a tight structure. The 30% and 60%-γ-PFGA materials contained different proportions of FG and Bz, leading to heterogeneity and repulsion. In general, suitable size, negative zeta potential, and small PDI indicated that the CS NPs were stable and could achieve the EPR effect 36. The similar drug-loading capacity, nearly 100% encapsulation efficiency (Table S2) which resulted from π-π stacking between GA and the core material 46 and good stability in several matrices (Figure S6), showed the stabilization of GA by CS NPs.

### Reduction-activated charge conversion and drug release

The novel functional CS NPs underwent reduction-activated degradation followed by the consequent charge conversion and drug release. To assess their reduction-responsiveness, the size and morphology changes of different types of CS NPs incubated with 10 mM GSH (to mimic the tumor bioreductive environment) were observed by DLS and TEM. With the incubation time increasing, a division of the size distribution appeared, with larger sizes becoming more prevalent (Figure 2B). More intuitively, in Figure 2C, the upper row shows the initial states of the four types of CS NPs, spherical core-shell NPs. The middle and lower rows showed the morphology changes of the C_F0%_S and C_F100%_S NPs. For the C_F0%_S NPs, some degradation of the shells could be observed after 1 h. After 3 h, the shell was completely disassembled and only the core structure remained. Unlike other types of γ-PFGA, since the core of the GA/C_F0%_S NPs was non-reduction-sensitive, the degradation rate slowed markedly after the HA-SS-ATRA shell had been disassembled. C_F100%_S NPs were taken as representative of others owning a reduction-sensitive core. After 1 h, more of the shell degraded and core leakage could already be observed. Little core structure remained after 3 h. The drug release profiles in PBS and GSH (Figure 2D-E) were also consistent with the above results. In 10 mM PBS, all kinds of GA/CS NPs showed a similarly controlled drug release less than 20% within 72 h. In 10 mM GSH, an incremental increase in release was observed on going from GA/C_F0%_S NPs to GA/C_F100%_S NPs. The *t*_1/2_ of drug release was significantly different between GA/C_F0%_S NPs and the other types (*p*<0.001) (Figure S7) due to non-reduction-sensitivity of the 0%-γ-PFGA.

Another important characteristic of the functional CS NPs was their concurrent reduction-activated charge conversion (Figure 2F). To exclude the influences of the negatively charged HA-coated shell and the amphiphilic ionic GSH on the determination of zeta potential, DTT, an organic reducing agent 48, was used instead of GSH and incubated with the cores of CS NPs prepared from different types of γ-PFGA. After 3 h, the higher the grafting rate of the FG was, the more pronounced the charge-conversion ability the core materials would acquire (0%-γ-PFGA showed non-charge-conversion ability). Once the FG was exposed to a reducing environment, the disulfide bond was cleaved and the thiazole ring was formed through ring-closing metathesis (Scheme 1). As a consequence, positively charged N^+^ gradually accumulated and a stable charged structure was formed, which was confirmed by ^1^H-NMR (Figure S8). Compared to those at 0 h, the ^1^H-NMR spectra of core materials incubated with GSH for 1 h and 3 h featured a stronger new peak at δ=10.00 (s, -C**H**=N) and weaker peaks at δ=7.75 (s, FG-C**H**O) and 0.83 ppm (t, -CH_2_C**H**_3_), consistent with the cleavage of the disulfide bond and the formation of N^+^.

To our knowledge, in general, more positively charged species should exhibit stronger attraction to negatively charged cell membranes and nuclei. Thus, our functional CS NPs would be expected to show an increasing ability to penetrate between cells as the grafting rate of FG increased. This is quite important for deep tumor treatment 8. To think it over, in our drug delivery system, there seems to exist a conflict between tumor penetration and drug release. The more positive charge CS NPs obtain, the more promptly the material will disassemble, resulting in rapid drug release in the superficial zone of the tumor. Coincidently, both rapid drug release and deep penetration are two attributes required for different stages of tumors, laying the foundation for our idea to design a stage-specific cancer therapy strategy. For an early-stage tumor, rapid drug release is beneficial for strong damage. For an advanced-stage tumor, more of the drugs are expected to be withheld for transport to the interior zone. Thus, how to achieve the optimal balance and select the best type of drug-loaded carrier for penetration require further comparative bioactivity tests * in vitro* and * in vivo*.

### * in vitro* cytotoxicity, intracellular and intercellular delivery

**Figure 3:**
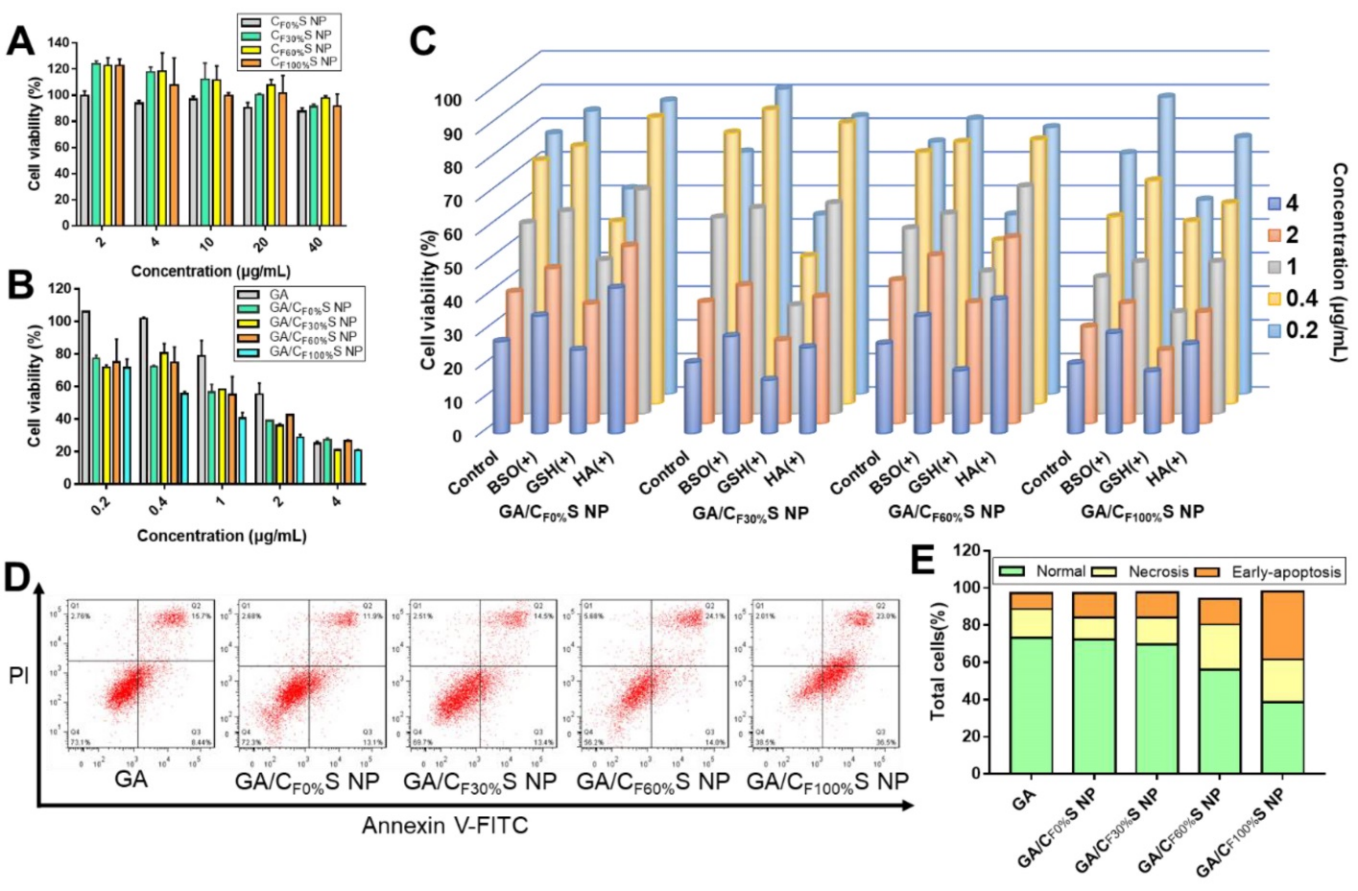
** (A)** Cytotoxicity of different types of CS NPs to L02 cells for 24 h.** (B)** Cytotoxicity of different types of GA/CS NPs to HepG2 cells for 24 h. **(C)** Cytotoxicity of different types of GA/CS NPs to HepG2 cells for 24 h with BSO, GSH and HA pretreatment. **(D)** Flow cytometry analysis of cell apoptosis after different formulations treatment (GA concentration of 1 μg/mL) using annexin V-FITC/PI assay. **(E)** Statistical analysis of normal, necrotic and early-apoptotic cells.

Cytotoxicity was evaluated by an MTT assay. Different types of blank CS NPs at relevant doses showed no obvious cytotoxicity towards L02 cells, a kind of normal liver cells (Figure 3A and Table S3). This guaranteed the biosafety of our CS NPs. For HepG2 tumor cells, the different types of GA/CS NPs all enhanced cell death, especially at low drug concentrations (Figure 3B). The IC_50_ values of the four types of GA/CS NPs were all less than 1.3 μg/mL, among which GA/C_F100%_S NPs considerably reached 0.62±0.02 μg/mL, significantly lower than that of GA (2.42±0.29 μg/mL) (Table S4). The factors underpinning this enhanced cytotoxicity may include the following: 1) The stabilization effect of CS NPs on GA. GA is easily metabolized by biological matrices 49, which caused the destruction of its 9,10 carbon-carbon double bond, a crucial site for its antitumor activity 50. Hence, it is important to deliver GA into cells before it is degraded. 2) HA-CD44 receptor mediated endocytosis. CD44 receptors are expressed on many tumor cells 37, including HepG2 cells, and show a high affinity towards HA (our shell material HA-SS-ATRA). 3) GSH induced rapid intracellular drug release. It is well known that there is a high level of GSH in tumor cells 26,51. It serves to cleave disulfide bonds in both the core and shell materials, thereby accelerating the drug release.

To confirm the above potential mechanism, several control groups were examined. HepG2 cells were pretreated with 50 mM BSO for 12 h (a GSH biosynthesis inhibitor) 52, 10 mM GSH for 2 h (to increase the concentration of intracellular GSH) and 2 mg/mL HA for 2 h (to saturate CD44 receptors). Compared to a blank control group, concentration-dependent decreases in cytotoxicity were observed for the groups pretreated with BSO and HA, and an increase for that pretreated with GSH (Figure 3C), as expected.

**Figure 4:**
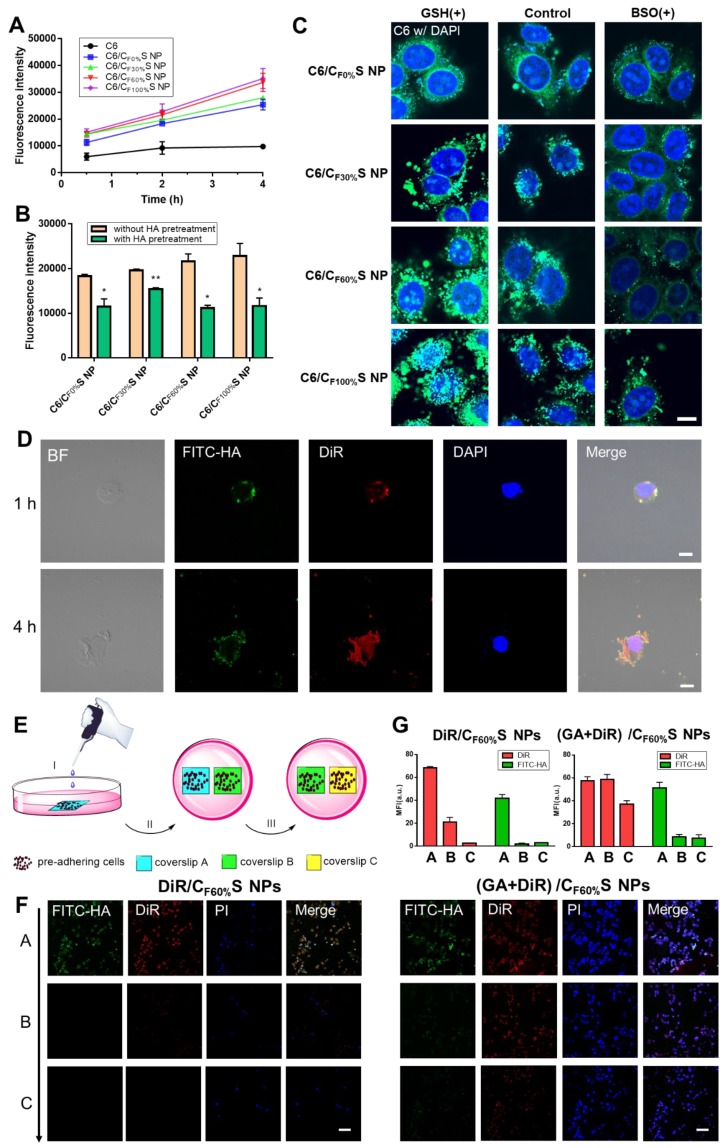
** (A)** Cellular uptake of HepG2 cells incubated with different types of C6/CS NPs for 0.5, 2 and 4 h measured by flow cytometry.** (B)** Cellular uptake of HepG2 cells incubated with different types of C6/CS NPs with/without HA pretreatment for 2 h measured by flow cytometry. *p<0.05, **p<0.01 compared to without HA pretreatment. **(C)** Nuclei targeting of different types of C6/CS NPs in HepG2 cells for 4 h of incubation with GSH or BSO pretreatment observed by confocal laser scanning microscopy. The nuclei were stained by DAPI (blue) and C6 was green. Scale bars indicate 10 µm. **(D)** Intracellular shell detachment from the core of (GA+DiR)/C_F60%_S NPs incubated for 1 and 4 h. The shell of HA-SS-ATRA was labeled with FITC (green). The nuclei were stained by DAPI (blue) and DiR was red. Scale bars indicate 10 µm. **(E)** Schematic of two-dimensional intercellular delivery experiment. (I) incubate coverslip A with (GA+DiR)/C_F60%_S NPs for 6 h. (II) co-culture coverslip A with B for another 12 h. (III) co-culture coverslip B with C for another 12 h. **(F)** Two-dimensional intercellular delivery of FITC-labeled DiR/C_F60%_S NPs and (GA+DiR)/C_F60%_S NPs. The nuclei were stained by PI (blue) and DiR was red. Scale bars indicate 100 µm. **(G)** Mean fluorescence intensity of FITC or DiR in each coverslip.

To assess cellular internalization of the drug, C6 was used in place of GA for fluorescence analysis. Quantitative analysis of cellular uptake by flow cytometry showed an obvious increase in C6/CS NPs compared to C6 (Figure 4A). Furthermore, HA pretreatment to saturate CD44 receptors partially blocked the uptake of C6/CS NPs (Figure 4B). All of the results supported the interpretation that the enhanced cytotoxicity of GA/CS NPs could be ascribed to more drug uptake and release.

To further investigate differences in the cytotoxicity of the four types of GA/CS NPs, cell apoptosis analysis using annexin V-FITC/PI staining was performed (Figure 3D-E). With the grafting rate of FG increasing, the total amount of necrosis and apoptosis cells (Q2+Q3) increased (from 25.0% to 58.5%) compared to GA group (24.1%). Interestingly, 36.5% early-apoptosis cells (Q3) were observed in GA/C_F100%_S NPs group. This suggested that our GA/CS NPs might induce apoptosis through GSH depletion as GA/C_F100%_S NPs depleted more intracellular GSH at the same dose 53,54. Moreover, due to the reduction-activated charge conversion, the nuclei-targeting abilities of different C6/CS NPs were not identical (Figure 4C). GSH and BSO pretreatments were also applied to validate the mechanism. No obvious differences of the fluorescence intensity were observed between the control, GSH and BSO pretreatment groups for C6/C_F0%_S NPs due to non-reduction-sensitivity of the core material. For the other three types, the fluorescence intensities were in the order of GSH (+) > control > BSO (+) due to the decrease of reduction-activated degradation and drug release. With C6/C_F100%_S NPs, numerous green fluorescent dots were seen inside and outside of the nuclei, indicating a superior nuclei-targeting ability compared to the other groups. The stronger cytotoxicity may be attributed to multiple antitumor mechanisms involving GA being operative in the nuclei 39-41.

To further assess the fate of our GA/CS NPs following intracellular and intercellular delivery, we used FITC-labeled HA-SS-ATRA to prepare (GA+DiR)/C_F60%_S NPs for representation of the core and shell individually. Figure 4D shows that after incubation for 1 h the core and shell were almost overlapped around the nucleus, indicating uptake of the whole CS NPs. At 4 h, the fluorescence of DiR was released and became distributed throughout the whole cell, indicating core-shell separation due to intracellular reduction-activated degradation. The core NPs, a positively charged transition state, as confirmed in Figure S8, finally targeted the nuclei. Notably, the cells were seen to decrease in size and undergo apoptosis due to the effect of GA, providing the potential for penetration of the remaining drug-loaded core NPs.

Next, a two-dimensional intercellular delivery experiment was conducted (Figure 4E) 9. PI was used to label apoptotic cells. From Figure 4F, three conclusions can be drawn. 1) For (GA+DiR)/C_F60%_S NPs, the fluorescence of FITC decreased quickly in coverslip B and C, while DiR achieved good intercellular delivery (Figure 4G), meaning that the shell was mostly detached in the coverslip A and the core showed the penetration ability mediated by electrostatic interactions. 2) the fluorescence of PI was obvious in coverslip A, indicating extensive cell apoptosis, which provided a necessary condition for the remaining core NPs to escape and penetrate. 3) For DiR/C_F60%_S NPs group, we could see that without the cytotoxicity effect of GA, the cell apoptosis hardly occurred, and the fluorescences of FITC and DiR were both weak in the coverslip B and C (Figure 4G). It can be explained that the positively charged core NPs were mostly trapped in the live cells so that they could not support further intercellular delivery. This corroborated our delivery mode dependent on the apoptosis of superficial cells. Furthermore, * in vitro* 3D tumor spheroid and * in vivo* tumor penetration experiments were also conducted (Figure S9). Similar results were obtained, implying that with the depth increasing, the fluorescence of FITC still remained in the outer space, PI showed a strong intensity in the superficial layer, and DiR achieved good penetration.

As far, the intracellular and intercellular delivery mode of our GA/CS NPs could be summarized as follows: 1) activated by intracellular reduction environment, 2) the process depends on the damage effect of the drug on the tumor cells; 3) drug-loaded positively charged core NPs are responsible for the delivery. This lays the important foundation for our tumor penetration study.

### Tumor penetration

**Figure 5:**
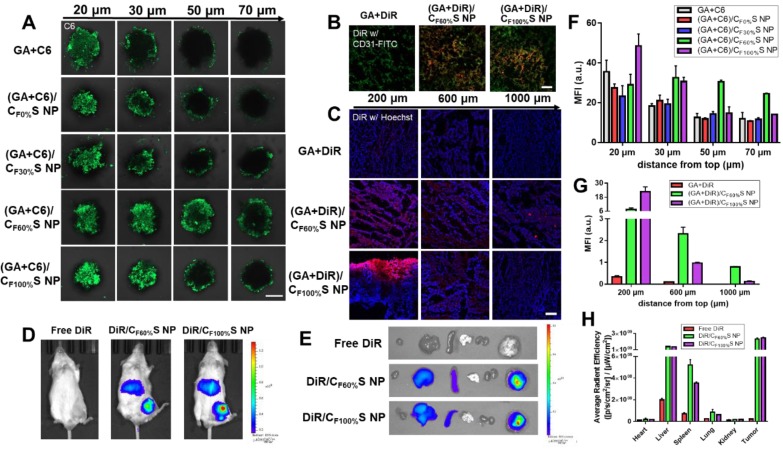
** (A)** Confocal images of 3D tumor spheroids with different treatments for 6 h at different distances from top. C6 was green. Scale bars indicate 200 µm. **(B)** Confocal images of the tumor sections harvested from the mice following intravenously injection for 24 h. The blood vessels were stained by FITC-CD31 antibody (green) and DiR was red. Scale bars indicate 100 μm. **(C)** *in vivo* penetration into the tumors of the Heps-bearing mice after intratumor injection for 24 h. The frozen tumor sections were observed at different depths below the injection site using CLSM. The nuclei were stained by Hoechst (blue) and DiR was red. Scale bars indicate 100 μm. **(D)** The * in vivo* imaging of tumor-bearing mice after intravenously injection for 8 h. **(E)** *Ex vivo* imaging of the major organs. **(F)** Mean fluorescence intensity of the 3D tumor spheroids with different treatments at different distances from top. **(G)** Mean fluorescence intensity of the frozen tumor sections with different treatments at different depths below the injection site. (H) Average radiant efficiency of the organs measured by *ex vivo* ROI imaging analysis.

The above antitumor activity research from the two-dimensional perspective showed that GA/C_F100%_S NPs had the strongest cytotoxicity due to the rapidest drug release. Then, to further evaluate the drug-loaded penetration abilities of different types of GA/CS NPs, a three-dimension tumor spheroids model was established and C6 was added with GA to mark the location of the drug. After treatment for 6 h, the fluorescence intensity attenuated with the increasing distance from top (Figure 5A), which was further analyzed semi-quantitatively (Figure 5F). Among these, the fluorescence of GA+C6 quickly disappeared due to lack of charge-conversion-induced penetration ability and lower cellular uptake. At the same distance of 20 μm, the strongest fluorescence intensity of (GA+C6)/C_F100%_S NPs was found due to their rapidest reduction-activated degradation of the carrier, indicating burst-like drug release and excellent damage potential to the superficial tumor cells. However, it was quite obvious at the distance of 50 and 70 μm that only (GA+C6)/C_F60%_S NPs showed an outstanding fluorescence, which meant that there existed a balance point that only an appropriate degree of degradation could allow enough drugs to penetrate into deep cells. Otherwise, irrespective of the charge-conversion ability, most of the drug was released too soon. Based on the above studies, the functional CS NPs identified as valuable for further research were thus GA/C_F60%_S and GA/C_F100%_S NPs.

Prior to evaluation of the penetration ability in a solid tumor, * in vivo* tumor-targeting ability was first studied using DiR dye as a marker. After intravenous injection for 8 h, free DiR was widely distributed around the body and quickly eliminated. In comparison, DiR/C_F60%_S NPs and DiR/C_F100%_S NPs showed good tumor-targeting efficiency, despite partial liver uptake (Figure 5D). The *ex vivo* main organs were also imaged (Figure 5E) as well as ROI analysis (Figure 5H). The results showed that the fluorescence intensity of DiR/C_F60%_S NPs and DiR/C_F100%_S NPs were 10.0- and 10.6-fold higher, respectively, than that of free DiR, and nearly 52% and 57% of the available DiR was targeted to tumor sites compared to 7% of free DiR. As is well known, HA-related nano preparations show good active targeting abilities to tumor, albeit with partial capture by the reticuloendothelial system (RES), including the liver, spleen and other immune organs 19,55. Thus, it was necessary to evaluate whether GA/CS NPs would have an impact on these organs. The drug-vascular distribution in the tumor sites after intravenously injection for 24 h also told the similar results (Figure 5B).

For a more direct comparison of their penetrations in solid tumors, GA+DiR and their CS NPs were intratumorally injected at the same depth. After 24 h, the fluorescence at different depths below the injection site (Figure 5C) and a semi-quantitative analysis (Figure 5G) revealed a similar trend as in * in vitro* 3D tumor spheroids. GA+DiR showed little fluorescence owing to lack of cellular uptake and penetration. At 200 μm, (GA+DiR)/C_F100%_S NPs displayed intense and widespread fluorescence. The fluorescence decreased sharply with increasing depth. In contrast, (GA+DiR)/C_F60%_S NPs showed a slower and smoother decrease in fluorescence intensity with depth, indicating a superior drug-loaded penetration ability, making it more suitable for deep tumor treatment.

Hence, two types of functional GA/CS NPs were selected for the following pharmacodynamic research * in vivo*: GA/C_F60%_S NPs, with the best drug-loaded penetration ability, and GA/C_F100%_S NPs, with the rapidest reduction-responsive drug release.

### Antitumor activity of the stage-specific administration strategy

**Figure 6:**
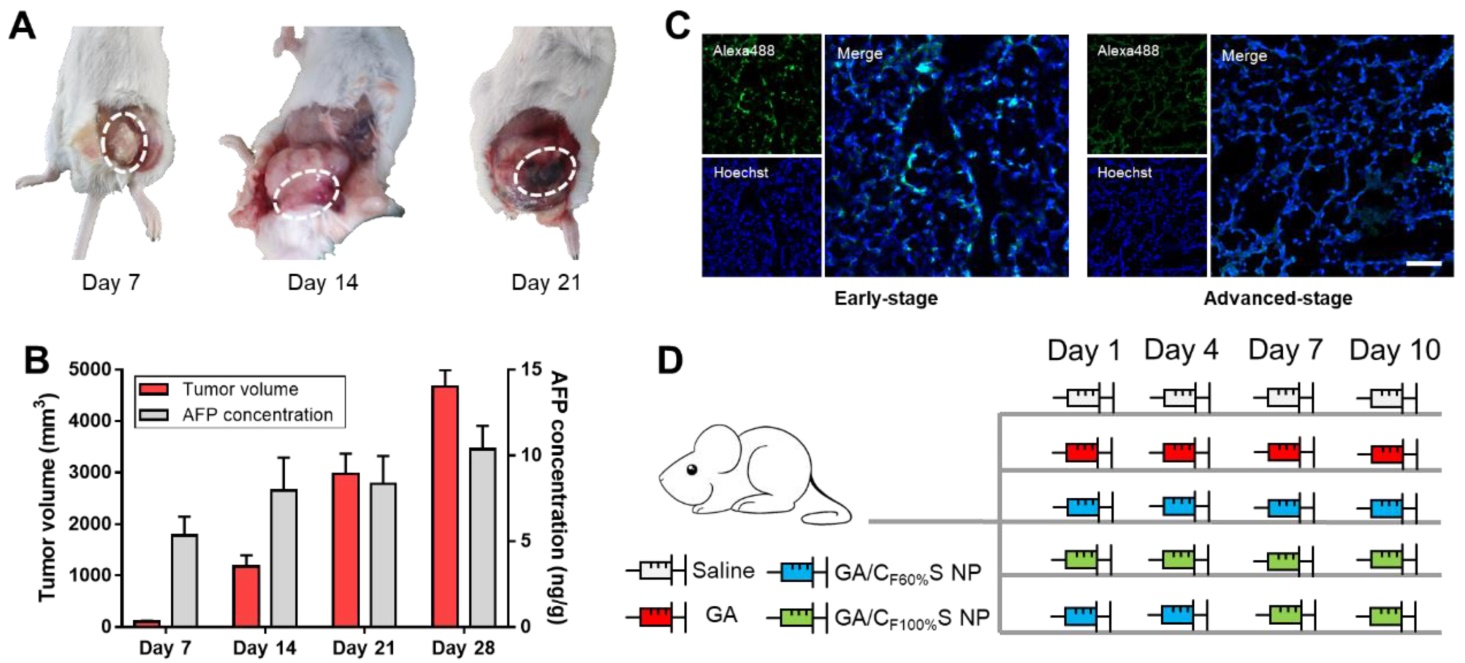
** (A)** Anatomical observation of Heps tumor at different days after subcutaneous injection at the left leg. The white dotted circles indicate the features of each stage.** (B)** Tumor volume and AFP concentration of mice at different days after subcutaneous injection at the left leg. **(C)** Cell proliferation activity of the center of the tumors in different stages determined by EdU assay. The EdU was labeled with Alexa488 (green), and the nuclei was labeled with Hoechst (blue). Scale bars indicates 100 μm. **(D)** Administration routines of five groups for comparison. The same dose was 6 mg/kg for GA.

To make full use of the two types of functional CS NPs in accordance with the concept of precision medicine, we sought to devise a stage-specific administration strategy for the best antitumor efficacy. To this end, a two-stage hepatocellular carcinoma model (murine Heps tumor), including early and advanced stages, was established according to the TNM staging criteria assisted with alpha-fetoprotein (AFP) detection. Hepatocellular carcinoma is the third most common malignancy around the world, and the 5-year survival rate is less than 5% 56,57. Besides, AFP, a classic hepatocellular carcinoma biomarker 58,59, is well defined as a regulatory factor in the growth, recurrence and mortality of hepatocellular carcinoma 60. Thus, it is essential to choose the most effective treatment strategy for different stages of hepatocellular carcinoma in terms of chemotherapy. After subcutaneous injection of Heps cells, tumor volume measurement with anatomical observation and AFP concentration assay were conducted at the different growth stages (Figure 6A-B). At day 7, the tumor was small (about 100 mm^3^), soft, sparse and easy to separate from other nearby tissues, and could thus be recognized as the early stage. At day 14, the tumor was large (about 1,000 mm^3^), hard and showed obvious adhesions with nearby muscle tissue. At day 21, the tumor got even larger (about 3,000 mm^3^) and vascular invasion had appeared. These tissue and vascular invasions could be categorized as proximal transfer, corresponding to “regional nodes metastasis” of TNM. Until day 30, no distant metastasis or ascites were seen, which might be associated with the tumor type. Meanwhile, the AFP concentration in tumor homogenates collected on different days also showed a gradual increase, reflecting the tumor progression. Therefore, through tumor size combined with their corresponding anatomical features and AFP levels, two stages of Heps were established for the following research 61. One was the early stage (day 7, tumor volume ≈ 100 mm^3^), and the other was the advanced stage (day 14, tumor volume ≈ 1,000 mm^3^). Besides, cell proliferation activity at the center of the tumors was also determined by EdU assay (Figure 6C). The green fluorescence of Alexa488 was stronger in the early stage, indicating that tumor cells proliferated more quickly than those in the advanced stage. This could be attributed to the features of cancer stem cells, which were located more in the center of the tumor in the advanced stage than in the early stage, and could enter a dormant state as a self-protection measure due to lack of nutrition from blood vessels 11,62,63. After the establishment of the two-stage hepatocellular carcinoma, we evaluated the antitumor activity of our functional CS NPs by applying them in the stage-specific administration strategy (Figure 6D).

**Figure 7:**
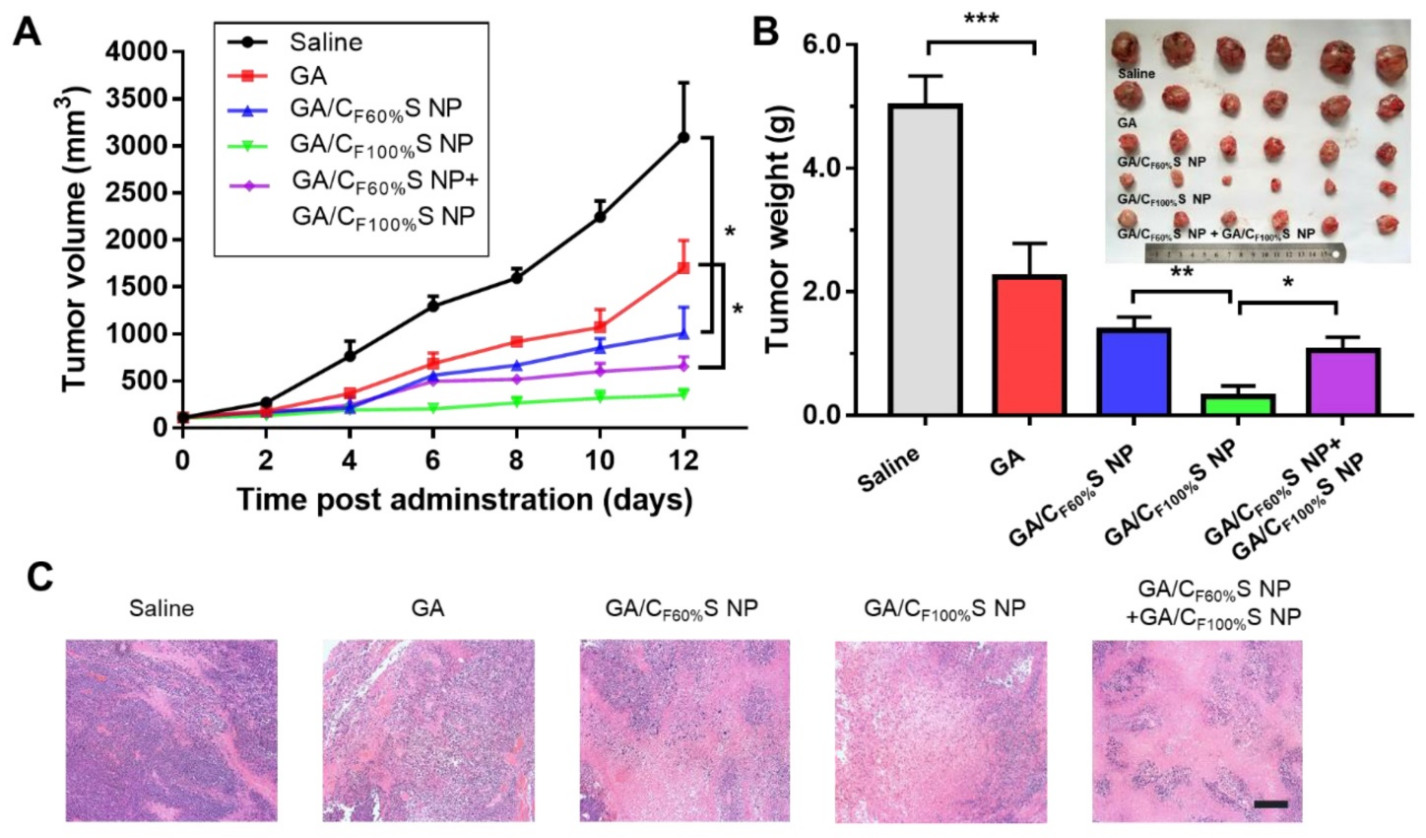
*** in vivo* antitumor efficacy of the early-stage Heps tumor. (A)** Tumor growth curves of different groups after treatment following the predetermined administration routines every three days. *p<0.05.** (B)** Tumor weights and photographs of the treatment groups after sacrifice. *p<0.05, **p<0.01, ***p<0.001.** (C)** Histological analyses of tumor sections after various treatments by H&E staining. Scale bars indicate 200 μm.

For the early-stage tumor, GA/C_F100%_S NPs showed a prominent tumor inhibition ability (about 94%) compared to GA (about 54%), GA/C_F60%_S NPs (about 73%), and GA/C_F60%_S NPs + GA/C_F100%_S NPs (about 79%) (Figure 7B). These results could be easily understood because, as mentioned above, GA/C_F100%_S NPs showed the strongest antitumor efficacy, which was sufficient for the tumor of small size in the early stage. From the plot of the tumor volume (Figure 7A), it can be seen that for GA/C_F60%_S NPs + GA/C_F100%_S NPs, the results were similar to that for GA/C_F60%_S NPs over the first 6 days; once injected with GA/C_F100%_S NPs, the efficacy improved swiftly. H&E staining also indicated that more tumor cells were damaged by GA/C_F100%_S NPs (Figure 7C).

**Figure 8:**
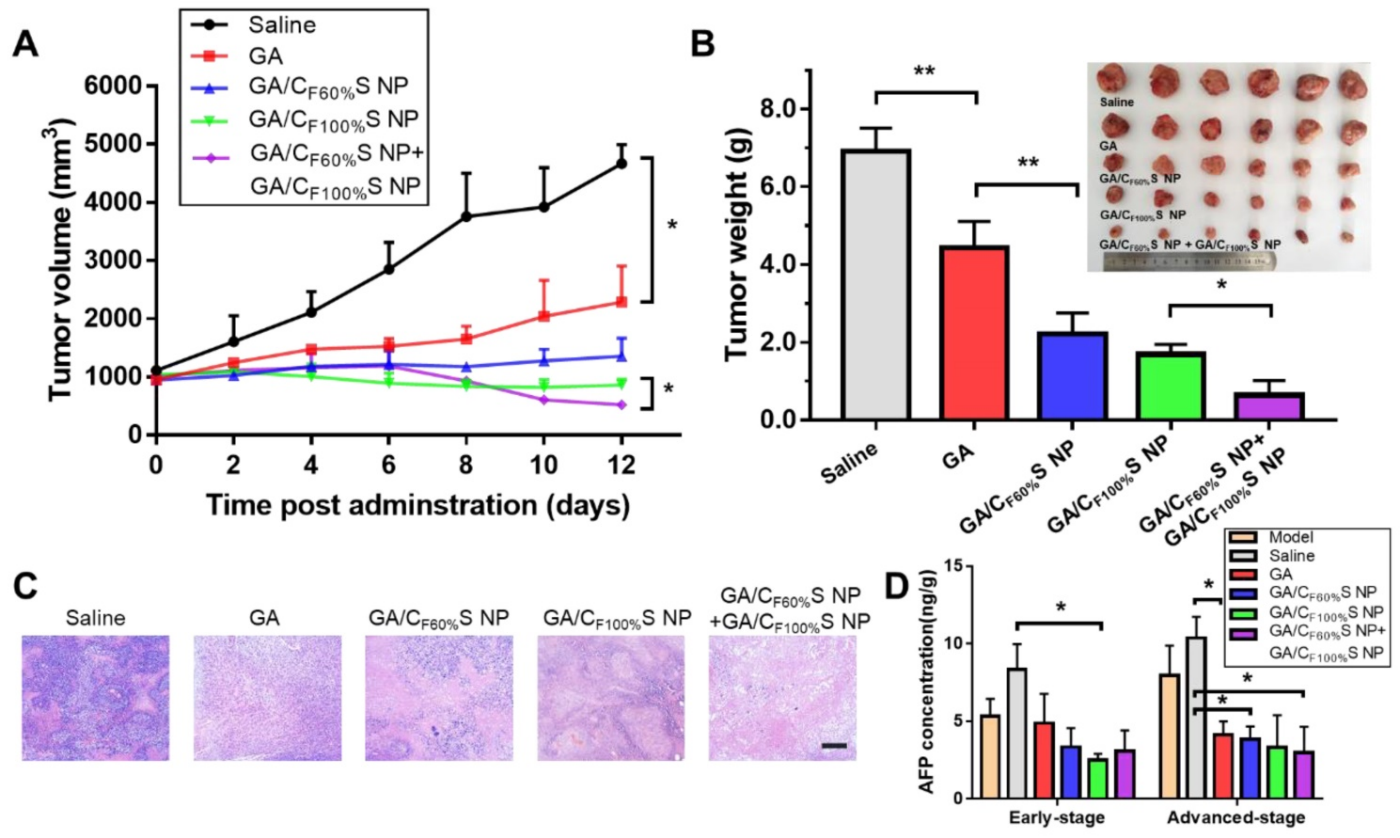
*** in vivo* antitumor efficacy of the advanced-stage Heps tumor. (A)** Tumor growth curves of different groups after treatment following the predetermined administration routines every three days. *p<0.05. **(B)** Tumor weights and photographs of the treatment groups after sacrifice. *p<0.05, **p<0.01. **(C)** Histological analyses of tumor sections after various treatments by H&E staining. Scale bars indicate 200 μm. **(D)** AFP concentration determination in tumor homogenate of different groups at different tumor stages. *p<0.05.

For the advanced-stage tumor, the excellent tumor inhibition trend for GA/C_F60%_S NPs + GA/C_F100%_S NPs (about 90%), compared to those for GA (about 35%), GA/C_F60%_S NPs (about 68%) and GA/C_F100%_S NPs (about 75%), was clearly evident (Figure 8B). What made a difference was that GA/C_F60%_S NPs + GA/C_F100%_S NPs showed a quite strong efficacy when finishing the injection of GA/C_F60%_S NPs and beginning to inject GA/C_F100%_S NPs (Figure 8A). These results demonstrated the effectiveness of our “Punch-Damage” strategy. In detail, GA/C_F100%_S NPs were initially somewhat advantageous over GA/C_F60%_S NPs; the former, with stronger antitumor efficacy, could damage the superficial tumor cells more quickly and cause layer by layer destruction. When it comes to GA/C_F60%_S NPs, despite their superior penetration ability, they had insufficient power to clear the superficial tumor cells completely, so the apparent tumor volume remained larger. The most striking result was that with GA/C_F60%_S NPs + GA/C_F100%_S NPs. A possible explanation was that GA/C_F60%_S NPs first charged ahead to punch a hole, opening the way for GA/C_F100%_S NPs to destroy the remaining tumor cells on a large scale. The same trends were also found by H&E staining (Figure 8C). Our results implied that this “punch-damage” strategy could reduce the tumor size more effectively and swiftly. It also implied that advanced-stage tumors needed drug penetration more than early-stage tumors. Notably, the AFP concentration in tumor homogenates, detected for validation of our treatment efficacy (Figure 8D), showed a stage-related trend, suggesting that it might be viewed as a supplementary factor to the traditional staging criteria 64. After treatment, this index showed a significant decrease, reminding us to pay attention to its change as a preliminary indicator of tumor recurrence.

In a preliminary safety evaluation, insignificant differences in the body weights and immune organ coefficients of mice were found during treatment in both two-stage models (Figure S10). H&E staining of the normal organs after such administration revealed no obvious toxic effects (Figure S11). Blood routine and blood biochemistry examinations revealed no significant differences between groups, apart from slight increases in ALT and AST in the GA group (Table S5), indicating slight hepatotoxicity of free GA. Fortunately, GA/CS NPs did not elicit such damage.

Based on the above results, the potential advantages of our functional materials may be threefold: 1) It may avoid the risk of tolerance and safety when increasing the dose for advanced-stage tumors in the routine chemotherapy; 2) During multiple dosing, similar materials could cause receptor saturation, whether in RES or the related HA receptors in other tissues 65. With the sensitivity of these receptors suppressed, subsequently introduced similar CS NPs could escape the capture and reach the tumor site more efficiently. 3) Due to the balance between penetration and drug release of these types of materials, the penetration ability of which stems from carrier degradation, we believe that our two-stage administration strategy is superior to those involving only one type of particle-supported drug.

## Conclusions

In summary, a novel series of reduction-activated charge-conversional core-shell nanoparticles with different abilities has been developed for stage-specific cancer chemotherapy. TDS-grafted γ-PFGA was employed as the core material and HA-coated conjugate as the shell material. The prepared nanoparticles showed excellent capabilities of reduction-activated drug release and charge-conversion-induced deep tumor penetration. On the basis of two types of functional CS NPs, namely GA/C_F100%_S NPs, with the strongest reduction-responsive drug release ability, and GA/C_F60%_S NPs, with the strongest drug-loaded penetration ability, we proposed a stage-specific administration strategy against a two-stage hepatocellular carcinoma: GA/C_F100%_S NPs for the early-stage tumor, and sequential administration of GA/C_F60%_S NPs followed by GA/C_F100%_S NPs for the advanced-stage tumor. In particular, for deep penetration in an advanced-stage tumor, our “Punch-Destruction” strategy could elicit an efficient tumor inhibition as it optimally exploited the respective abilities of each type of CS NPs. It also makes sense that our CS NPs provide a novel platform for charge conversion activated by reduction. A future outlook of our study is to put it forward to the human cancer and combine with more clinical indicators for more practical application of precision medicine.

## Supplementary Material

Supplementary figures and tables.

## Abbreviations

BBB: blood-brain barrier
BSO: L-buthionine sulphoximine
Bz: benzyl
CTA: cetyltrimethylammonium
CS NPs: core-shell nanoparticles
CLSM: confocal laser scanning microscopy
DiR: 1,1-dioctadecyl-3,3,3,3-tetramethylindotricarbocyanine iodide
DLS: dynamic light scattering
DMF: N,N-dimethylformamide
DMSO: dimethyl sulfoxide
DTT: Dithiothreitol
EPR: enhanced permeability and retention
FCM: flow cytometry
FG: functional group
FITC: fluorescein isothiocyanate
GA: gambogic acid
GSH: Glutathione
HA-SS-ATRA: hyaluronic acid-all-trans retinoid acid with a disulfide bond as the linker
H&E: haematoxylin and eosin
MTT: 3-(4,5-dimethylthiazol-2-yl)-2,5-diphenyltetrazolium bromide
MWCO: molecular weight cut off
PDI: polydispersity index
γ-PFGA: poly (γ-glutamic acid) with different grafting rates of the FG
PI: propidiumiodide
RES: reticuloendothelial system
ROI: region-of-interest
TBA: tetra-n-butyl ammonium hydroxide
TDS: thiamine disulfide system
TEM: transmission electron microscopy
TNM: Tumor-Node-Metastasis
