# Supplementary material for: A stage-specific cancer chemotherapy strategy through flexible combination of reduction-activated charge-conversional core-shell nanoparticles
Source: Theranostics. 2019 Aug 21;9(22):6532–49. doi: 10.7150/thno.35057 (PMC6771249; doi:10.7150/thno.35057)
Supplement: Supplementary file 1 — Supplementary figures and tables. [file thnov09p6532s1.pdf]

Supporting Information

A stage-specific cancer chemotherapy strategy through flexible combination of reduction-activated charge-conversional core-shell nanoparticles

Lingfei Han[1], Yingming Wang[1], Xiaoxian Huang[2], Bowen Liu[2], Lejian Hu[2], Congyu Ma[1], Jun Liu[3], Jingwei Xue[3,4], Wei Qu[2], Fulei Liu[2,3,5*], Feng Feng[2,6*], Wenyuan Liu[1,7*]

1. Department of Pharmaceutical Analysis, China Pharmaceutical University, Nanjing 210009, China

2. Department of Natural Medicinal Chemistry, China Pharmaceutical University, Nanjing 210009, China

3. The Joint Laboratory of Chinese Pharmaceutical University and Taian City Central Hospital, Taian City Central Hospital, Taian, 271000, China

4. Taian City institute of Digestive Disease, Taian City Central Hospital, Taian, 271000, China

5. Pharmaceutical Department, Taian City Central Hospital, Taian, 271000, China

6. Jiangsu Food and Pharmaceutical Science College, Huaian 223003, China

7. Hangzhou Institute of Pharmaceutical Innovation, China Pharmaceutical University, Hangzhou 310018, China

*Corresponding author

*E-mail: liu_fulei@126.com.

* E-mail: fengsunlight@163.com.

* E-mail: liuwenyuan8506@163.com.

Methods

In vitro stability

The 900 μl of different types of CS NPs (100 μg/mL) were mixed with 100 μl of PBS (100 mM), fetal bovine serum (FBS) and incubated at 37 °C. At the predetermined time point, the size distribution was measured by DLS. Another 100 μl of GA and different types of GA/CS NPs (100 μg/mL) was mixed with 900 ul mice plasma and incubated at 37 °C. At the predetermined time point, the remaining GA was assayed by HPLC.

In vitro penetration and cell apoptosis of 3D tumor spheroids

The method was the same as that in *In vitro* tumor penetration in 3D tumor spheroids. The 3D tumor spheroids were incubated by FITC HA-labeled (GA+DiR)/$C_{F60\%}$S NPs for 6 h. The nuclei were stained by PI to detect the cell apoptosis.

In vivo penetration and cell apoptosis of Heps tumor

The method was the same as that in *In vivo* tumor penetration of intratumorally injected GA/CS NPs in tumor-bearing mice. the Heps-bearing mice were intratumorally injected with FITC HA-labeled (GA+DiR)/$C_{F60\%}$S NPs for 24 h. PI was injected *in situ* for 2 h before anatomy to stain apoptotic nuclei.

Preliminary safety evaluation

Male ICR mice (18~22 g) were divided into four groups (n = 6): Saline, GA/IS, GA/$C_{F60\%}$S NPs and GA/$C_{F100\%}$S NPs. The dosing interval and cycle were consistent to the method in *in vivo* anti-tumor activity. Two days after the last injection, blood

samples were collected for blood routine and blood biochemistry examination. The major organs (heart, liver, spleen, lung, kidney) were fixed in a 4% formaldehyde solution at room temperature for at least 48 h. H&E staining was performed and observed with an invert microscopy (CKX41, Olympus).

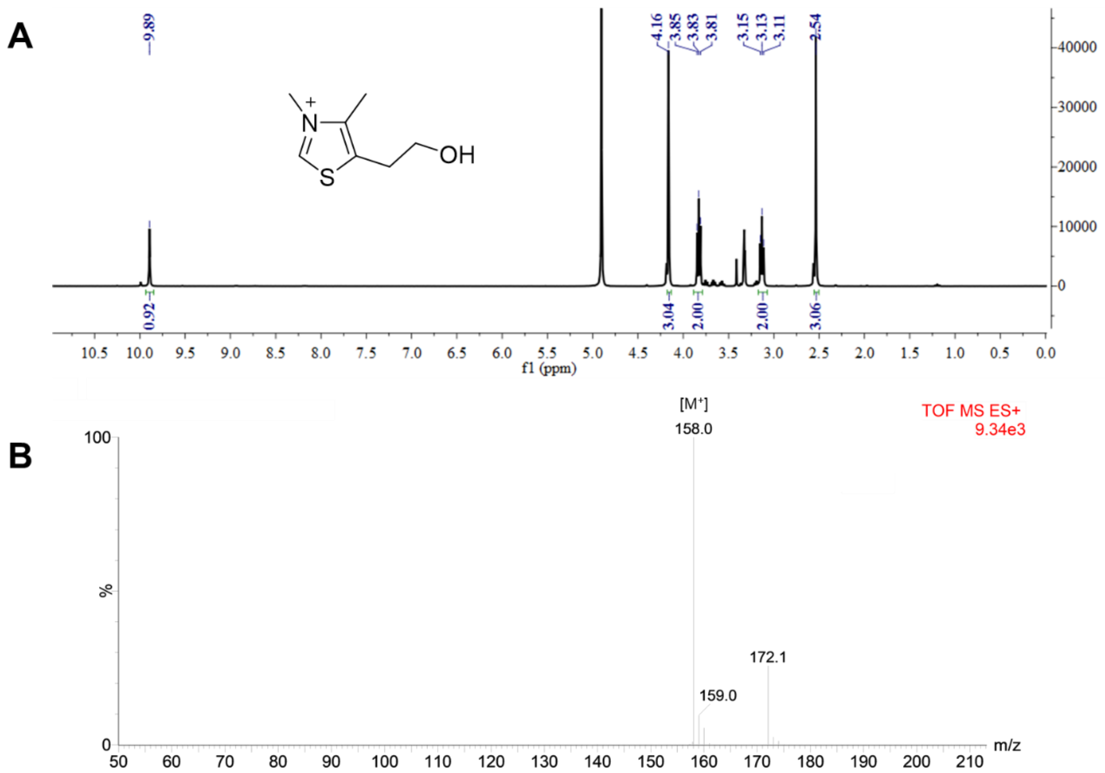

Figure S1. Characterization of compound 1. **(A)** 1H-NMR spectra. **(B)** Mass spectra.

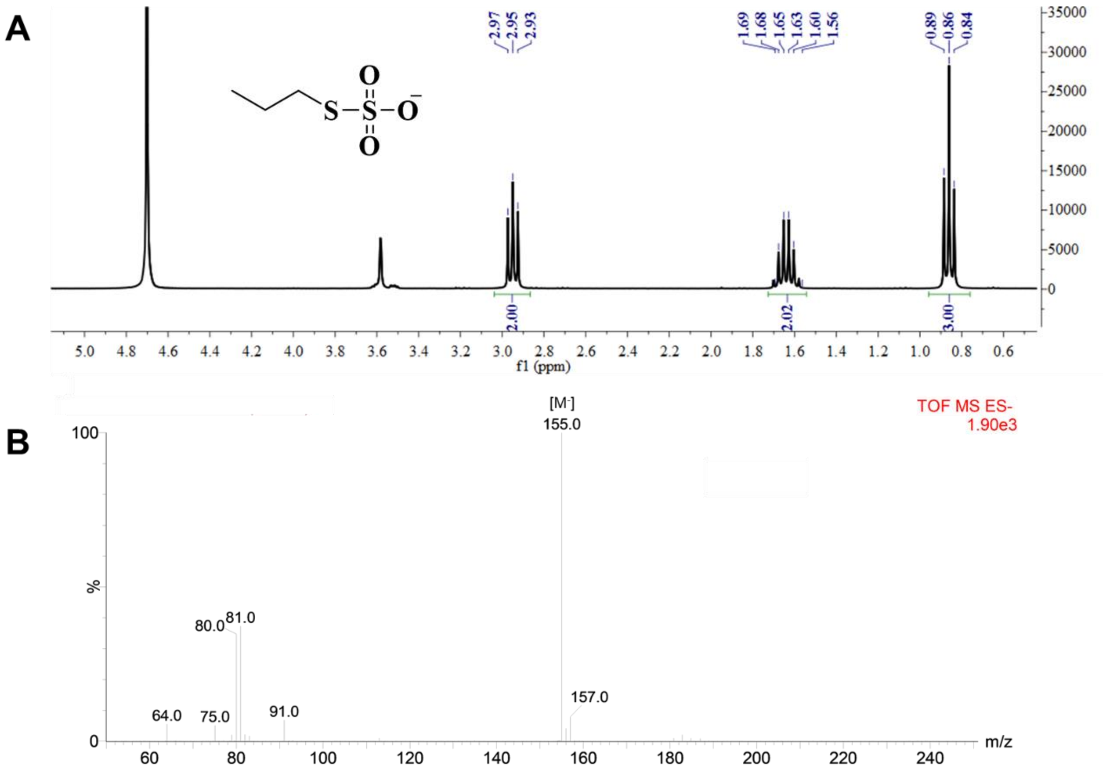

Figure S2. Characterization of compound 2. **(A)** ^{1}H-NMR spectra. **(B)** Mass spectra.

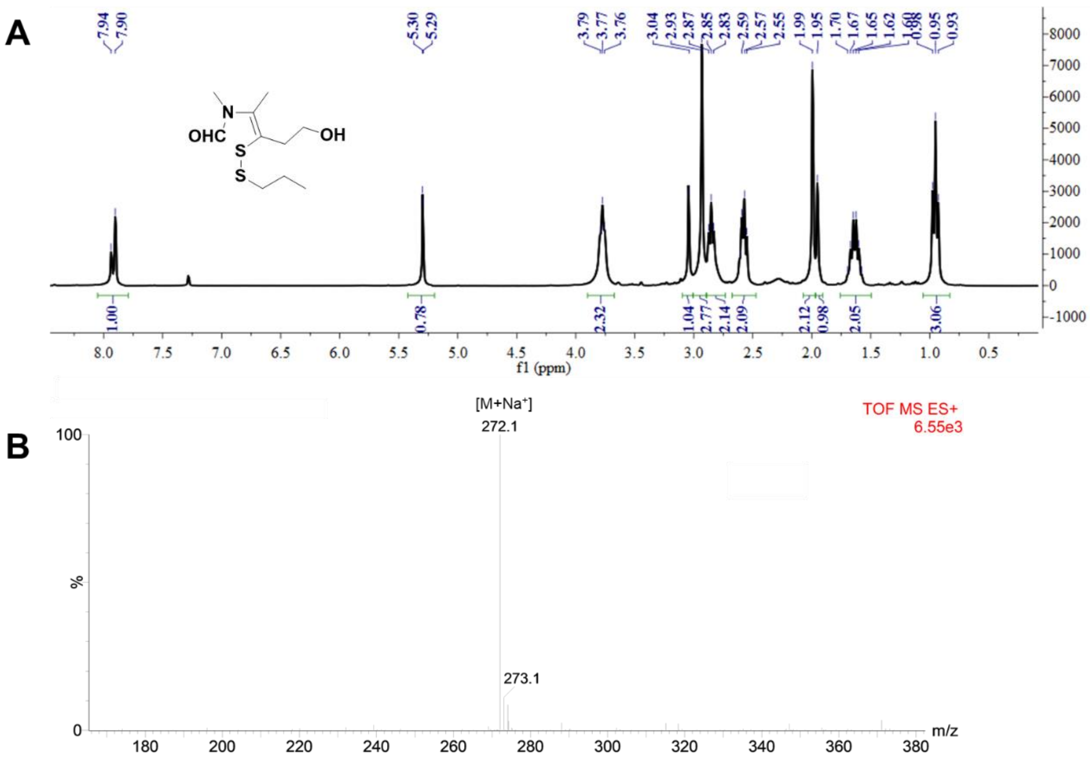

Figure S3. Characterization of compound 3. **(A)** ^{1}H-NMR spectra. **(B)** Mass spectra.

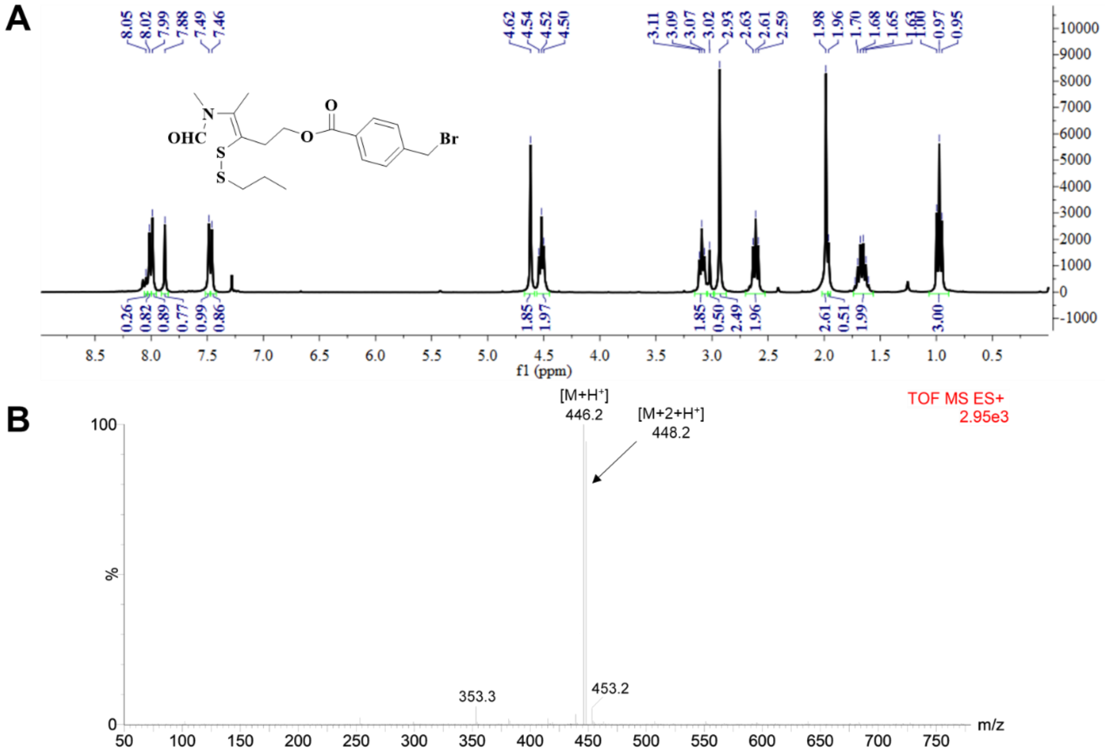

Figure S4. Characterization of compound 4. **(A)** ^{1}H-NMR spectra. **(B)** Mass spectra.

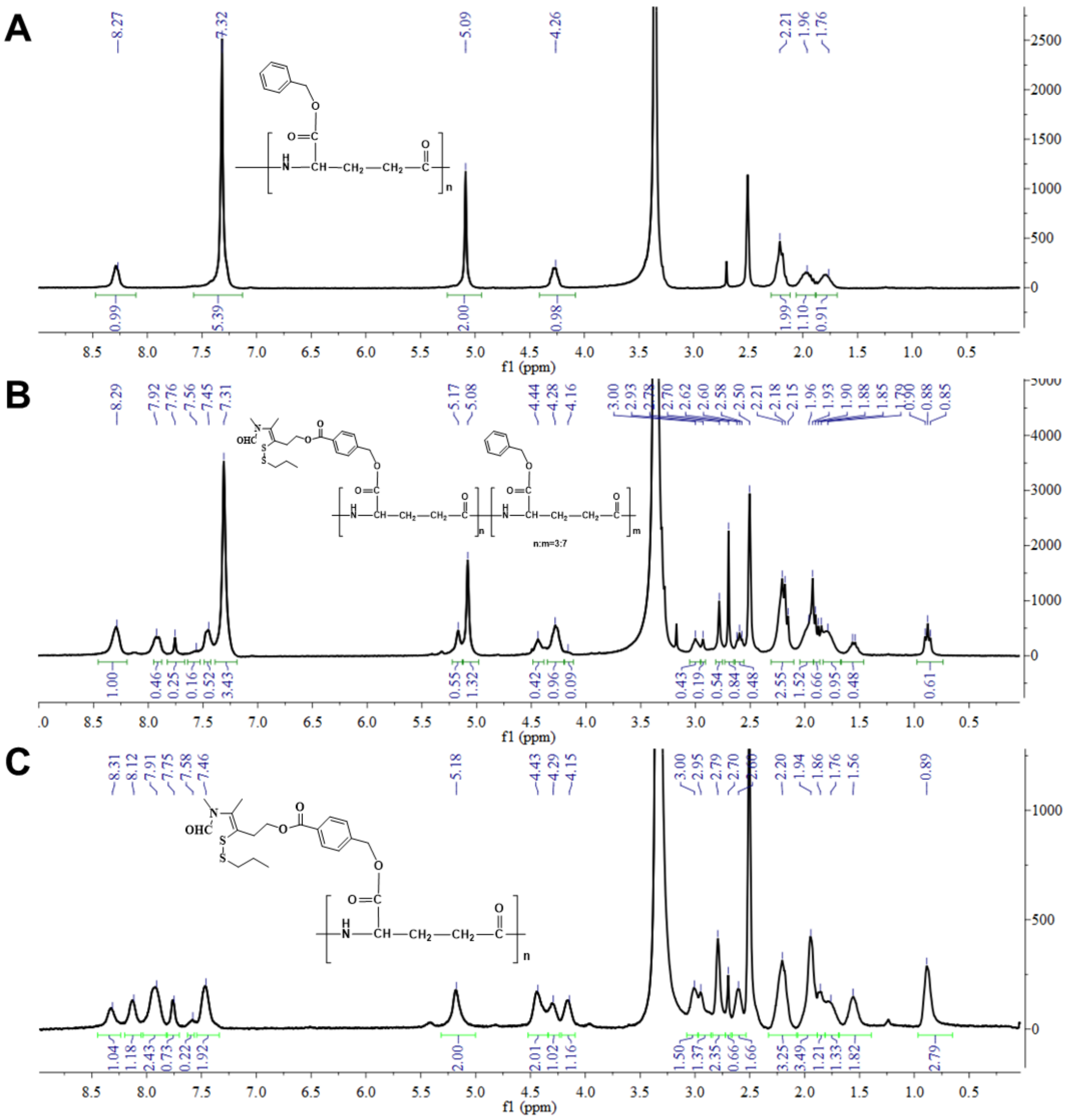

Figure S5. ^{1}H-NMR spectra of different types of γ-PFGA. **(A)** 0%-γ-PFGA. **(B)** 30%-γ-PFGA. **(C)**100%-γ-PFGA.

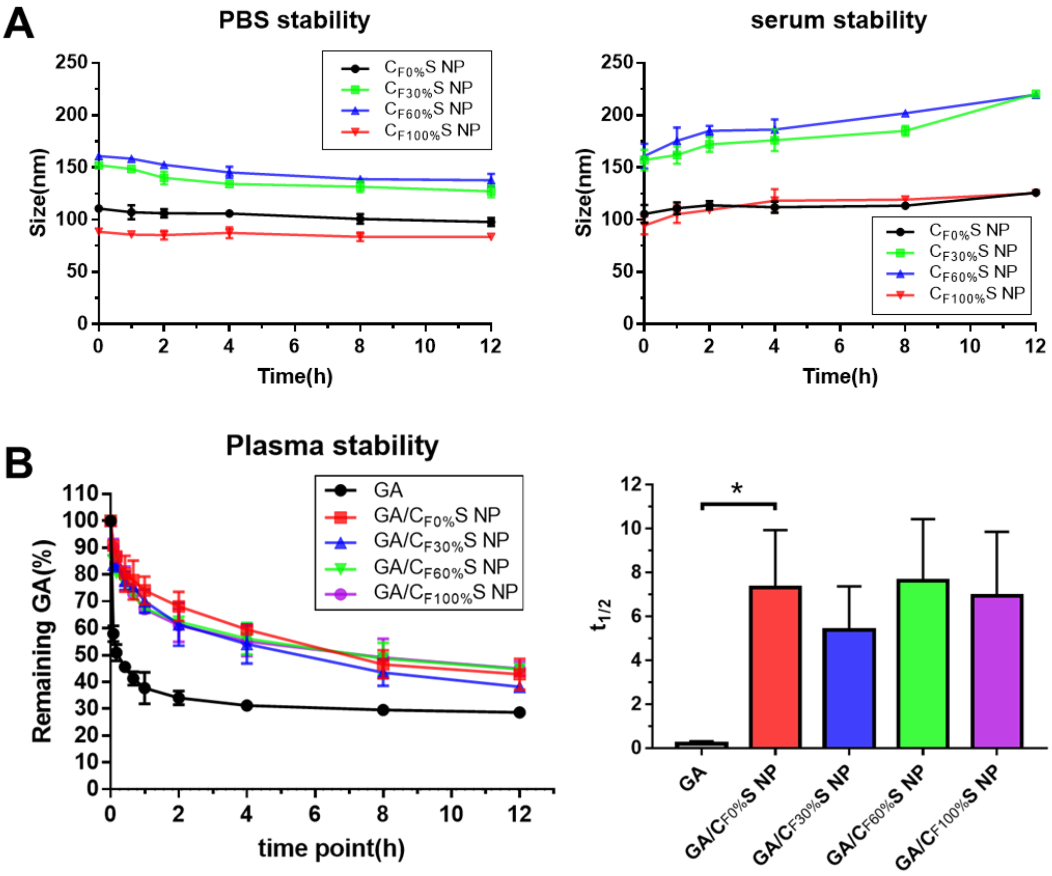

Figure S6. (A) Stability of different types of CS NPs incubated in PBS and serum. **(B)** Stability of different types of GA/CS NPs and GA incubated in plasma and the corresponding $t_{1/2}$. * p<0.05.

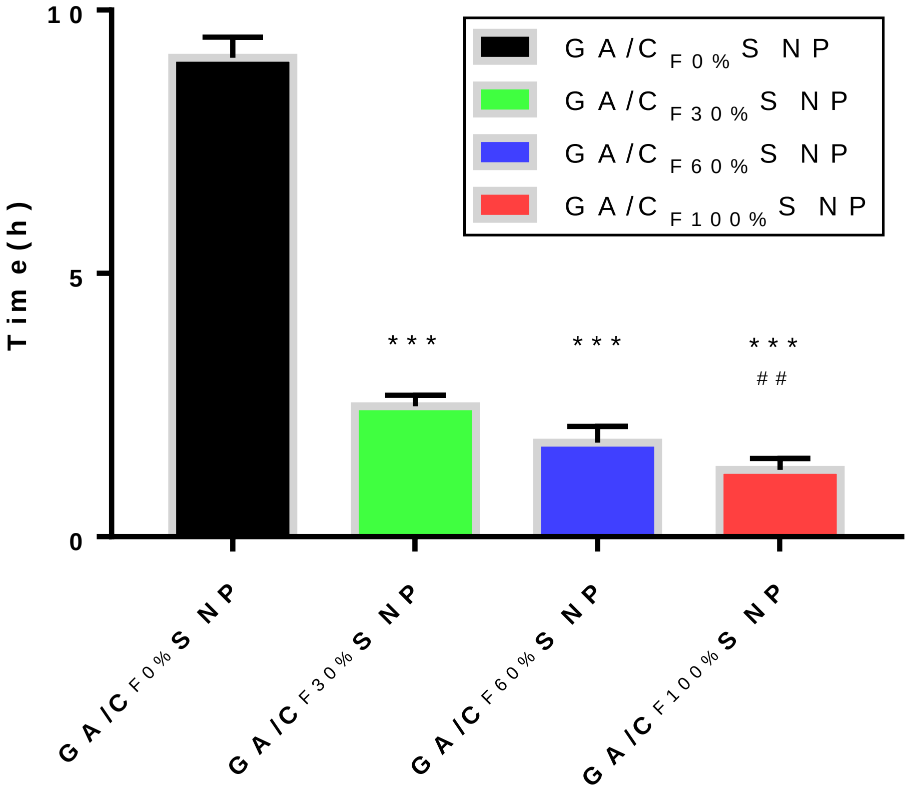

Figure S7. $t_{1/2}$ of drug release of different types of GA/CS NPs incubated in 10 mM GSH. *** $p<0.001$ (compared to GA/C$_{F0\%}$S NPs), $^{\#\#}$ $p<0.01$ (compared to GA/C$_{F30\%}$S NP).

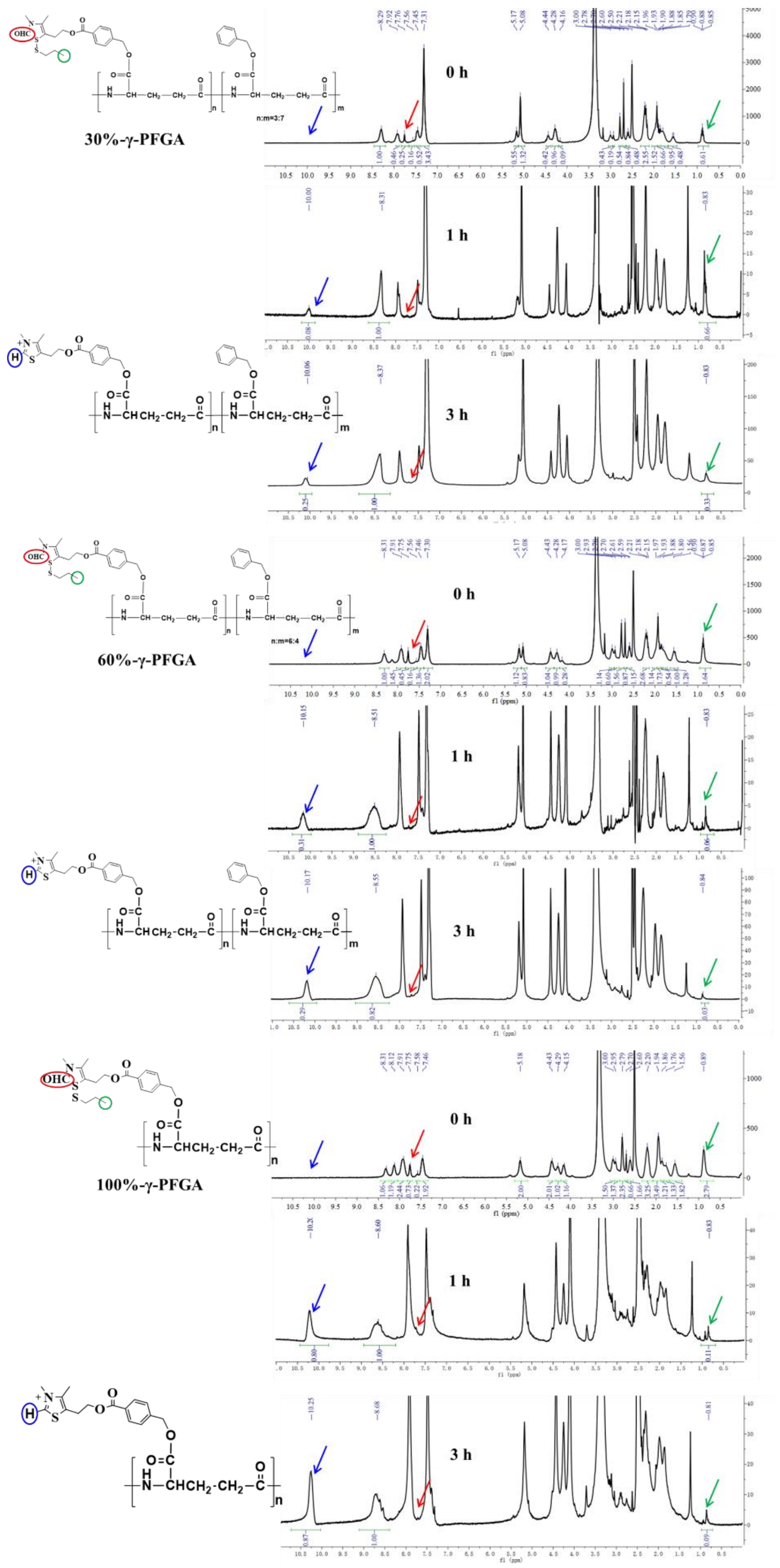

Figure S8. ^{1}H-NMR spectra of different types of core materials NPs incubated in 10 mM DTT for 0, 1 and 3 h.

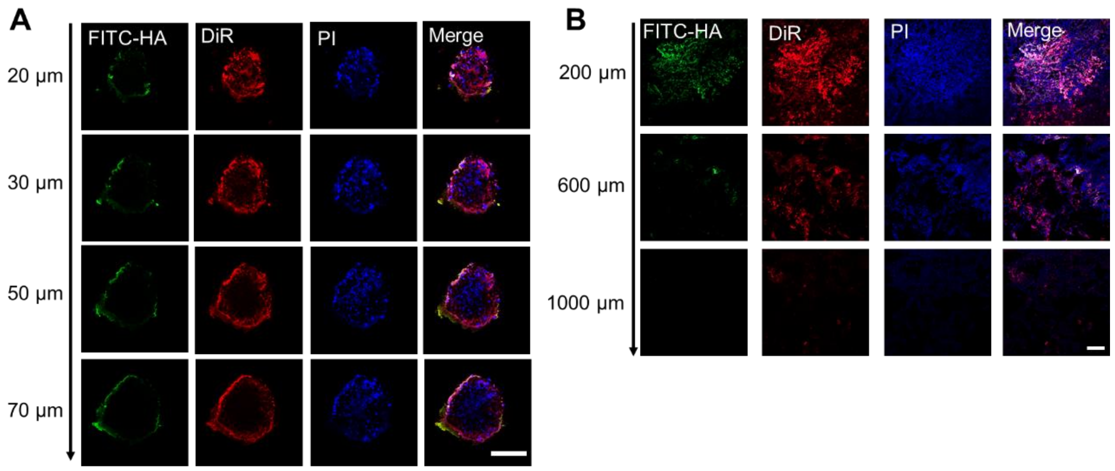

Figure S9. (A) Confocal images of 3D tumor spheroids incubated by FITC HA-labeled (GA+DiR)/$C_{F60\%}$S NPs for 6 h at different distances from top. The nuclei were stained by PI. Scale bars indicate 200 µm. **(B)** *In vivo* penetration into the tumors of the Heps-bearing mice after intratumor injection of FITC HA-labeled (GA+DiR)/$C_{F60\%}$S NPs for 24 h. PI was injected *in situ* to stain nuclei for 2 h before anatomy. The frozen tumor sections were observed at different depths below the injection site using CLSM. Scale bars indicate 100 µm.

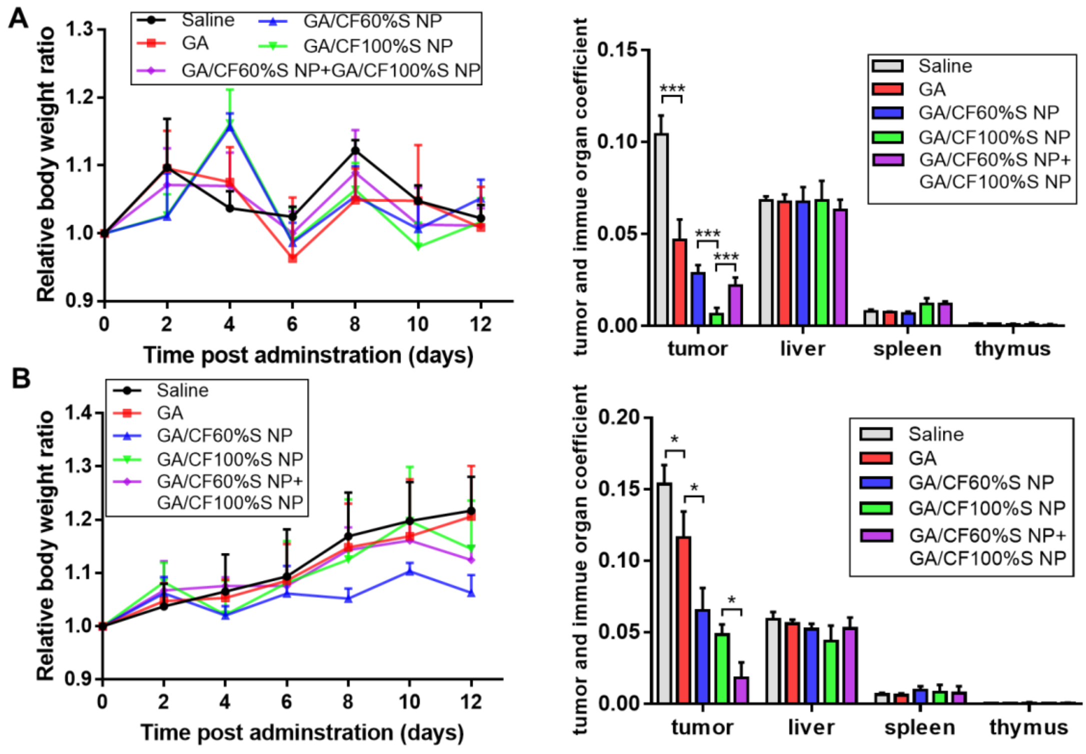

Figure S10. (A) Relative body weight ratio and tumor and immune organ coefficient of mice in early-stage with different treatment. *** $p<0.001$. **(B)** Relative body weight ratio and tumor and immune organ coefficient of mice in advanced-stage with different treatment. * $p<0.05$.

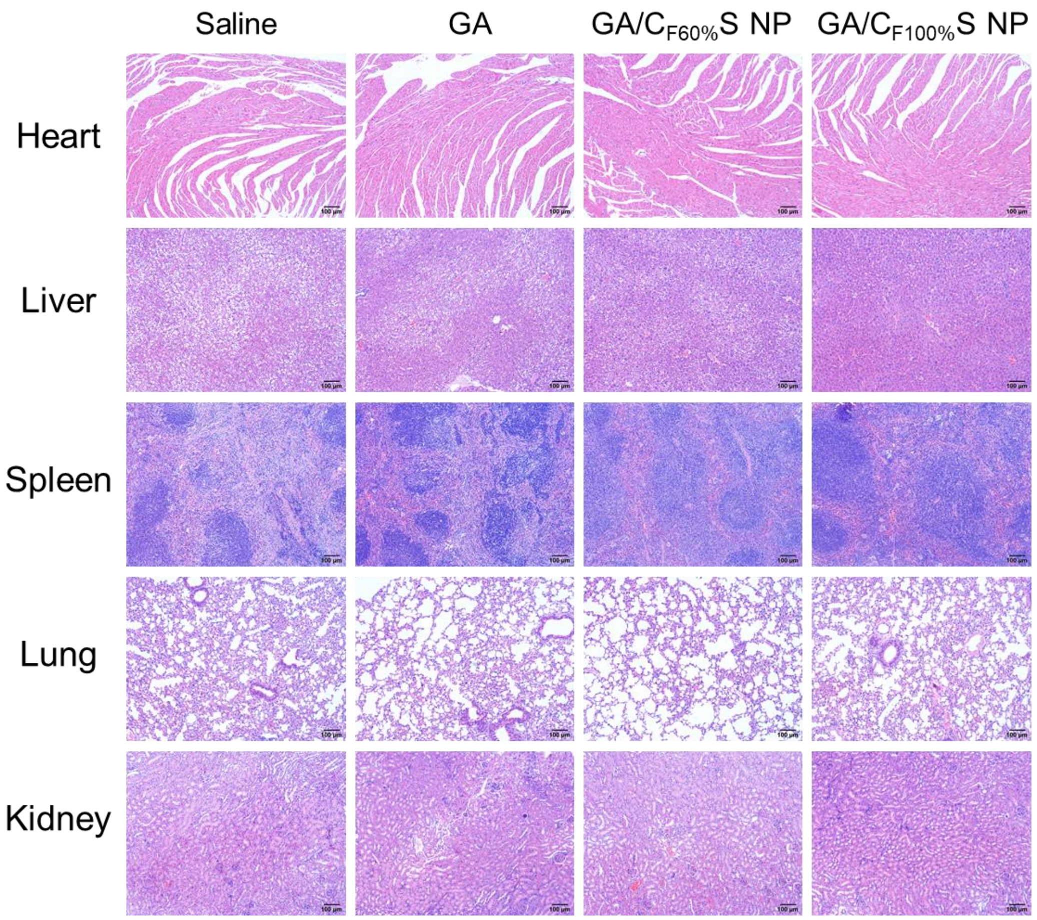

Figure S11. Histological assessment of the major organs (heart, liver, spleen, lung and kidney) by H&E staining. Scale bars indicate 100 μm.

Table S1. Calculation of different types of core materials.

Core material	Peak area (A)			Calculated grafting rate [a] (%)	Theoretical grafting rate (%)	Error [b] (%)
	FG(δ=5.17) PhC<u>H</u>$_2$	Bz(δ=5.08) C<u>H</u>$_2$Ar	γ-PGA(δ=8.30) N<u>H</u>			
0%-γ-PFGA	-	2.00	0.99	0	0	0
30%-γ-PFGA	0.55	1.32	1.00	29.41	30	-1.97
60%-γ-PFGA	1.12	0.83	1.00	57.44	60	-4.27
100%-γ-PFGA	2.00	-	1.04	96.15	100	-3.85

[a] Calculated grafting rate = $A_{FG}/(A_{FG}+A_{Bz}) \times 100\%$ (for 30% and 60%)

Calculated grafting rate = $1/2\ A_{FG}/A_{\gamma\text{-PGA}} \times 100\%$ (for 0% and 100%)

[b] Error = (Calculated grafting rate -Theoretical grafting rate)/Calculated grafting rate × 100%

Table S2. EE and DL of different types of GA/CS NPs.

	**EE/%**	**DL/%**
GA/$C_{F0\%}$S NP	89.34±0.88	6.20±0.06
GA/$C_{F30\%}$S NP	94.26±1.36	6.55±0.09
GA/$C_{F60\%}$S NP	98.79±0.09	6.86±0.01
GA/$C_{F100\%}$S NP	101.22±0.95	7.03±0.07

Table S3. *p* values analysis of cytotoxicity among different types of CS NPs to L02 cells for 24 h.

Concentration (μg/mL)	2	4	10	20	40
$C_{F0\%}$S NP% VS $C_{F30\%}$S NP	0.0106	0.1720	0.2779	0.3827	0.1778
$C_{F0\%}$S NP% VS $C_{F60\%}$S NP	0.0134	0.1659	0.2335	0.0628	0.1981
$C_{F0\%}$S NP% VS $C_{F100\%}$S NP	0.0087	0.5670	0.9812	0.3174	0.3904
$C_{F30\%}$S NP% VS $C_{F60\%}$S NP	0.9896	0.9755	0.9998	0.6584	0.9983
$C_{F30\%}$S NP% VS $C_{F100\%}$S NP	0.9916	0.7623	0.4879	0.9986	0.9723
$C_{F60\%}$S NP% VS $C_{F100\%}$S NP	0.9697	0.7483	0.4575	0.7478	0.9471

The *p* values show high levels of cell survival rate and no evident concentration-dependent cytotoxicity in four formulations groups.

Table S4. IC_{50} of different formulations to HepG2 cells.

	GA	GA/$C_{F0\%}$S NP	GA/$C_{F30\%}$S NP	GA/$C_{F60\%}$S NP	GA/$C_{F100\%}$S NP
IC_{50}(μg/mL)	2.42+0.29	1.22+0.01 **	1.18+0.00 **,#	1.25+0.01 **,$	0.62+0.02 **,###,$$$,&&&

***p*<0.05(compared to GA), #*p*<0.1, ###*p*<0.01(compared to GA/$C_{F0\%}$S NP), $*p*<0.1, $$$*p*<0.01(compared to GA/$C_{F30\%}$S NP), &&& *p*<0.01(compared to GA/$C_{F60\%}$S NP).

Table S5. Blood routine and blood biochemistry examination results (data are shown as mean ± SD, n = 6).

Group	wbc (10^9/L)	lym (10^9/L)	mon (10^9/L)	gra (10^9/L)	rbc (10^{12}/L)	hgb (g/L)	plt (10^9/L)
Saline	4.00±0.85	2.87±1.12	0.20±0.10	0.80±0.14	1.73±0.28	132.00±9.90	1683.33±550.35
GA	2.40±0.28	2.05±0.35	0.05±0.07	0.35±0.07	1.89±0.13	134.67±4.16	1693.00±722.66
GA/$C_{F60\%}$S NP	4.03±2.38	2.47±1.42	0.13±0.13	0.73±0.42	1.44±0.69	134.33±7.57	1680.50±887.53
GA/$C_{F100\%}$S NP	5.00±2.26	4.10±1.70	0.20±0.08	0.83±0.29	1.76±0.46	130.00±4.83	1721.00±176.78

Group	ALT	AST	TBIL	BUN	CREA
Saline	184.61±29.17	399.33±62.40	27.31±2.81	20.41±2.89	81.82±2.59
GA	252.38±66.02	582.91±13.08	31.06±4.39	23.48±2.01	84.48±5.45
GA/$C_{F60\%}$S NP	176.54±60.07	390.05±26.88	33.13±8.69	23.98±1.84	83.91±3.72
GA/$C_{F100\%}$S NP	174.58±33.38	468.82±89.76	19.68±0.63	22.80±3.02	80.88±3.81
